# Macrophage TRIM21 lactylation exacerbates infection-induced orchitis through enhancing STAT1-mediated CXCL9 and CXCL10 production

**DOI:** 10.3389/fimmu.2025.1684836

**Published:** 2026-01-14

**Authors:** Wenjing Tang, Wenjie Chen, Na Li, Wei Li, Zhigang Lei, Wenhui Sun, Xuan Xie, Yihong Jiang, Ying Chen, Lei Xu, Jifeng Zhu, Yalin Li, Jiahao Sha, Yang Dai, Sha Zhou, Xiaojun Chen, Chuan Su

**Affiliations:** 1 State Key Laboratory of Reproductive Medicine and Offspring Health, National Vaccine Innovation Platform of Nanjing Medical University, Key Laboratory for Pathogen Infection and Control of Jiangsu Province, Department of Pathogen Biology and Immunology, School of Basic Medical Sciences, Nanjing Medical University, Nanjing, Jiangsu, China; 2 Reproductive Medicine Center, Department of Obstetrics and Gynecology, Shanghai General Hospital, Shanghai Jiao Tong University School of Medicine, Shanghai, China; 3 Department of Laboratory Medicine, Nanjing First Hospital, Nanjing Medical University, Nanjing, Jiangsu, China; 4 State Key Laboratory of Reproductive Medicine, Nanjing Medical University, Nanjing, Jiangsu, China; 5 National Health Commission Key Laboratory of Parasitic Disease Control and Prevention, Jiangsu Provincial Key Laboratory on Parasite and Vector Control Technology, Jiangsu Provincial Medical Key Laboratory, Jiangsu Institute of Parasitic Diseases, Wuxi, Jiangsu, China; 6 Key Laboratory for Pathogen Infection and Control of Jiangsu Province, Department of Pathogen Biology and Immunology, School of Basic Medical Sciences, the Affiliated Wuxi People’s Hospital of Nanjing Medical University, Wuxi People’s Hospital, Wuxi Medical Center, Nanjing Medical University, Nanjing, Jiangsu, China

**Keywords:** CXCL9 and CXCL10, infection-induced orchitis, lactylation, macrophage, TRIM21

## Abstract

**Introduction:**

Infection-induced orchitis, a leading cause of acquired male infertility affecting 8%–12% of couples globally, is driven by unresolved inflammatory responses following bacterial infection.

**Methods:**

We employed uropathogenic *Escherichia coli* (UPEC)- and lipopolysaccharide (LPS)-induced orchitis models to define the mechanisms underlying testicular inflammation. We interrogated the cellular sources of CXCL9/CXCL10 and assessed macrophage-driven inflammatory cell recruitment and spermatogenic disruption. Mechanistic studies were focused on lysine lactylation, STAT1 protein stability, ubiquitin–proteasome–mediated degradation, and the role of the E3 ubiquitin ligase TRIM21.

**Results:**

We demonstrate that macrophages are the predominant source of CXCL9 and CXCL10 responsible for recruiting inflammatory cells into the testis, thereby disrupting spermatogenesis. Mechanistically, the lysine lactylation in macrophages promotes STAT1-mediated CXCL9 and CXCL10 expression by inhibiting ubiquitin–proteasome pathway-mediated STAT1 degradation. Specifically, K345 lactylation of the E3 ubiquitin ligase TRIM21 attenuates ubiquitin–proteasome pathway-mediated STAT1 degradation in macrophages by preventing its interaction with STAT1.

**Discussion:**

This study provides the first evidence that non-histone lactylation (TRIM21 K345) exacerbates inflammatory orchitis and highlights TRIM21 lactylation or CXCL9/10 as promising therapeutic targets for infection-associated male infertility.

## Introduction

Male infertility accounts for 30%–50% of all infertility cases, affecting approximately 8%–12% of reproductive-aged couples globally (1). Infections and the subsequent inflammatory responses in the genital tract are considered common causes of male infertility, second only to oligospermia (2). Although antibiotic therapy is the mainstay of clinical treatment for infection-induced orchitis, it fails to reverse inflammatory damage to epididymal and testicular function (3). Moreover, broad-spectrum anti-inflammatory agents, such as non-steroidal anti-inflammatory drugs and glucocorticoids, may impair the quantity and quality of the sperm and suppress testosterone production (4, 5). These limitations underscore the critical need to elucidate the immunopathological mechanisms underlying testicular inflammation for developing new therapeutic strategies.

Infection-associated orchitis is primarily caused by the gram-negative pathogens such as *Neisseria gonorrhoeae* and uropathogenic *Escherichia coli* (UPEC) (6). Emerging evidence supports that macrophages, principally from circulating monocytes, play key roles in the development and pathogenesis of infection-induced orchitis by mainly sensing LPS, a key virulence factor of gram-negative bacteria (7, 8). Notably, testicular macrophage infiltration correlates strongly with impaired spermatogenesis and infertility in clinical and experimental orchitis (9). However, the exact molecular mechanisms by which macrophages drive inflammatory testicular damage remain obscure.

Hypoxia and glycolysis are hallmarks of inflammation and generally considered to be crucial for the initiation and development of inflammatory responses in the context of multiple diseases, which subsequently increases the production and release of lactate (10, 11). Indeed, the extracellular lactate can be transported into cells such as macrophages and T cells and, as a consequence, affects the function of these cells (12, 13). Substantial evidence confirms that lactylation modification is a key component of lactate function and plays a pivotal role in modulating inflammation and other biological processes through inducing the lactylation of histones (14, 15) or non-histone proteins (16, 17). However, whether lactylation is involved in infection-induced orchitis remains unexplored.

In this study, we found that TRIM21 lactylation-mediated CXCL9 and CXCL10 production in a STAT1-dependent manner in macrophages promoted the inflammatory cell infiltration into the testis and consequently impaired spermatogenesis in UPEC- or LPS-induced orchitis. Mechanistic studies showed that K345 lactylation of TRIM21 inhibited TRIM21 binding to STAT1 to attenuate ubiquitin–proteasome pathway-mediated STAT1 degradation in macrophages. These results reveal a distinct mechanism underlying TRIM21 lactylation modulation of macrophage-mediated inflammation, thus providing potential therapeutic targets for the treatment of infection-induced orchitis.

## Results

### CXCL9 and CXCL10 contribute to inflammatory cell infiltration and impair spermatogenesis during infection-induced orchitis

To investigate the distribution of infiltrated immune cells in the testis during infection-induced orchitis, we employed UPEC- or LPS-treated mice to establish the models of infection-induced orchitis (18–20). UPEC or LPS was injected into the upper pole of the testes with a microsyringe, while vas deferens ligation was conducted after UPEC injection to prevent the spread of infection (**Supplementary Figures 1A, D**). The testes exhibited atrophy in mice treated with UPEC or LPS, concomitant with decreased testis weight and testis–body weight ratio (**Supplementary Figures 1B, C, E, F**). Histological analysis showed atrophic seminiferous tubules and damaged seminiferous epithelium in mice with UPEC- or LPS-induced orchitis (**Supplementary Figures 1G**, **2A–C**), accompanied by increased spermatogenic cell apoptosis (**Supplementary Figures 2D, E**). In addition, mice with orchitis exhibited breakdown of the blood–testis barrier, as indicated by reduced expressions of the Sertoli cell marker SOX9 and the tight junction protein ZO-1 (**Supplementary Figures 2F, G**). More importantly, UPEC infection or LPS treatment impaired spermatogenesis in mice, as shown by increased sloughed spermatogenic cells and malformed sperm (**Supplementary Figures 3A–E**) and reduced sperm motility (**Supplementary Figure 3F**). The impairment of spermatogenesis was associated with increased infiltration of immune cells, including macrophages, lymphocytes, and neutrophils, and elevated mRNA expression of pro-inflammatory cytokines including *Il-1β* and *Tnf-a* (**Supplementary Figures 4A–C**). Immunofluorescence analysis showed that macrophages were dramatically increased 5 days after the *in situ* injection of LPS (**Supplementary Figure 4D**). According to our gating strategy (**Supplementary Figure 5A**), flow cytometry analysis demonstrated that macrophages constituted the majority of immune cells during LPS-induced orchitis, as shown by the fact that macrophages, T cells, and dendritic cells account for 33.3%, 20.7%, and 17.2%, respectively (**Supplementary Figures 5B, C**).

**Figure 1:**
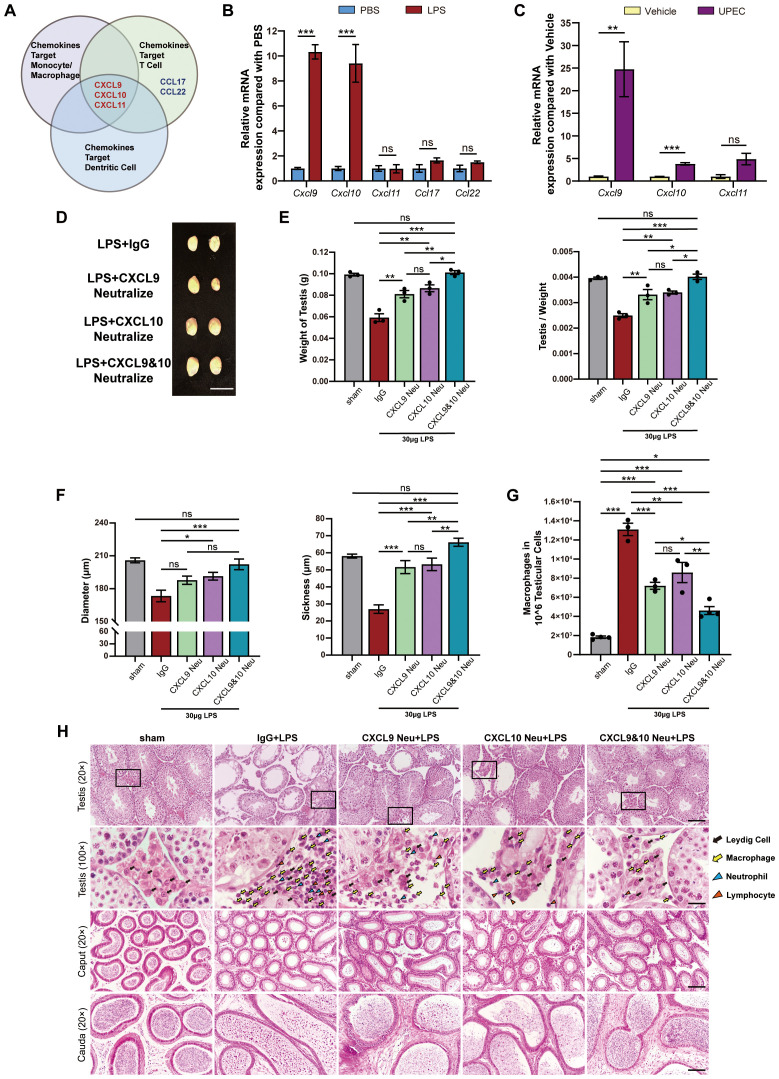
CXCL9 and CXCL10 contribute to inflammatory cell infiltration and impair spermatogenesis during infection-induced orchitis. **(A)** Venn diagram showing that chemokines CXCL9, CXCL10, and CXCL11 exhibit chemotactic effect on monocytes/macrophages, T cells, and dendritic cells, while CCL17 and CCL22 only target T cells. **(B)** Relative mRNA levels of *Cxcl9*, *Cxcl10*, *Cxcl11*, *Ccl17*, and *Ccl22* in the testes from LPS-induced orchitic and PBS control mice (*n* = 3). **(C)** Relative mRNA levels of *Cxcl9*, *Cxcl10*, *Cxcl11*, *Ccl17*, and *Ccl22* in the testes from UPEC-induced orchitic and vehicle control mice (*n* = 3). **(D)** Gross morphology of the testes from LPS-induced orchitic mice, orchitic mice with isotype IgG injection, and orchitic mice with single or dual neutralization of CXCL9 and CXCL10. Scale bar, 1 cm. **(E)** Weight and relative weight of the testes from the sham group of mice and aforementioned mice in **(B)** (*n* = 3). **(F)** Comparison of representative seminiferous tubules’ diameter and seminiferous epithelium thickness between the sham group and the above groups (*n* = 3). **(G)** Quantified flow cytometry showing the number of macrophages (CD45^+^CD11b^+^F4/80^+^) per 10^6^ testicular cells in the sham group and the above groups (*n* = 3–4). **(H)** Representative H&E staining images of seminiferous tubules, testicular interstitium, caput epididymis, and cauda epididymis from the indicated mice mentioned above. In the testicular interstitium, different arrows indicate different immune cells as shown. We rely on the characteristic nuclear morphology and localization of immune cells as a supportive, histological complement to the flow cytometric data in **(G)**. Scale bars, 100 μm at 20× magnification and 20 μm at 100× magnification. Two groups of data are statistically compared with Student’s *t-*test, while multiple groups of data are statistically compared with one-way ANOVA. Error bars represent mean ± SEM. *p < 0.05, **p < 0.01, ***p < 0.001; ns, not significant.

To investigate the mechanisms underlying immune cell infiltration into the testis during infection-induced orchitis, we evaluated the mRNA expressions of chemokines *Cxcl9*, *Cxcl10*, and *Cxcl11*, all of which could target macrophages, T cells, and dendritic cells for recruiting these cells into inflamed tissues (**Figure 1A**). As expected, the expression of *Cxcl9* and *Cxcl10* was substantially increased in the testis after UPEC or LPS treatment, while *Ccl17* or *Ccl22* exhibited no significant difference (**Figures 1B, C**). More importantly, neutralization of the chemokines CXCL9 and CXCL10 had an optimal role in alleviating testis atrophy (**Figures 1D, E**) and seminiferous tubule damage (**Figure 1F**), concomitant with reduced immune cell infiltration, including macrophages, lymphocytes, dendritic cells, and neutrophils (**Figures 1G, H**). As expected, neutralization of the chemokines CXCL9 and CXCL10 optimally ameliorated spermatogenesis, as reflected by increased testis morphology, weight, testis–body weight ratio, size, and spermatozoa, but decreased sloughed spermatogenic cells (**Figure 1H**). Taken together, these results indicate that CXCL9 and CXCL10 cause immune cell infiltration into the testis and impair spermatogenesis during infection-induced orchitis.

### Macrophages are a predominant cellular source of CXCL9 and CXCL10 in the testis during infection-induced orchitis

**Figure 2:**
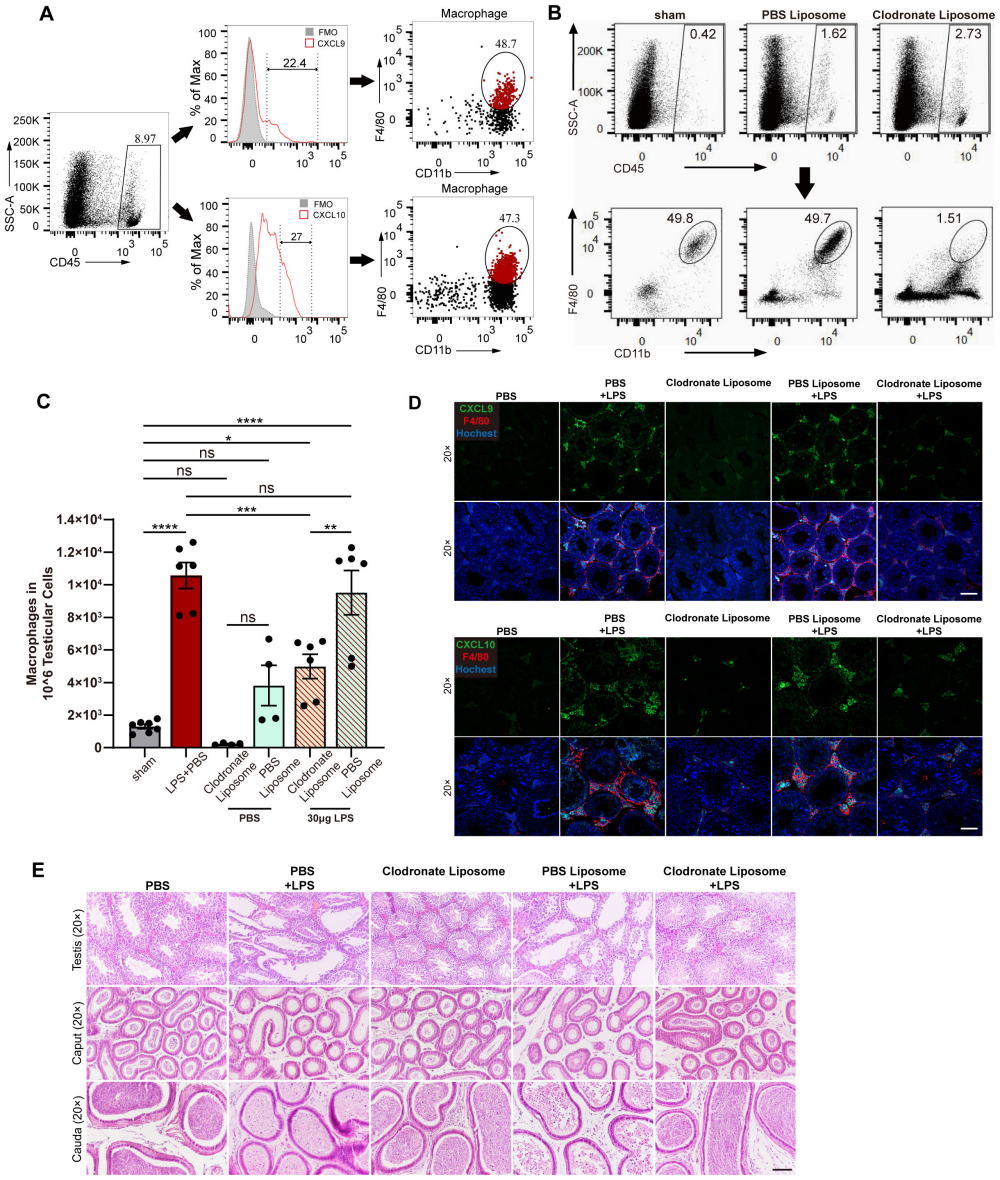
Macrophages are a predominant cellular source of CXCL9 and CXCL10 in the testis during LPS-induced orchitis. **(A)** Flow cytometry showing immune cells (gated on CD45^+^) expressing chemokines CXCL9 or CXCL10 in the testes from LPS-induced orchitic mice, with the proportion of macrophages identified. **(B)** Flow cytometry showing the proportion of testicular macrophages 3 days after *in situ* injection of clodronate liposomes in the testes. PBS liposomes were injected as control, while the sham group served as the negative control. **(C)** Quantified flow cytometry showing the number of macrophages (CD45^+^CD11b^+^F4/80^+^) per 10^6^ testicular cells in LPS-induced orchitic mice and sham/orchitic mice with *in situ* injection of clodronate liposomes/PBS liposomes. The sham group of mice served as the negative control. Sample sizes: sham = 7; LPS + PBS = 6; clodronate liposome + PBS = 4; PBS liposome + PBS = 4; clodronate liposome + LPS = 6; PBS liposome + LPS = 6. **(D)** Representative immunofluorescence images of the testes showing the co-localization of F4/80^+^ macrophages and chemokines CXCL9 or CXCL10 in mice with PBS/LPS/clodronate liposomes/PBS liposomes + LPS/clodronate liposomes + LPS intratesticular injection. Scale bar, 100 μm at 20× magnification. **(E)** Representative H&E staining images of seminiferous tubules, caput epididymis, and cauda epididymis from the indicated mice mentioned in **(D)**. Scale bar, 100 μm at 20× magnification. Data are statistically compared with one-way ANOVA. Error bars represent mean ± SEM. *p < 0.05, **p < 0.01, ***p < 0.001, ****p < 0.0001; ns, not significant.

To determine the major cellular source of CXCL9 and CXCL10 in the testis during infection-induced orchitis, we performed flow cytometry and found that macrophages accounted for approximately 50% of both CXCL9^+^ cells and CXCL10^+^ cells in the testis (**Figure 2A**). Furthermore, the increase in the production of CXCL9 and CXCL10 was abrogated after the elimination of macrophages in the testes by injecting clodronate liposomes *in situ* (**Figures 2B–D**). As expected, clodronate liposome treatment also improved spermatogenesis, as evidenced by increased spermatozoa and reduced sloughed spermatogenic cells in the caput epididymis and cauda epididymis (**Figure 2E**). Taken together, these results show that macrophages are a major cellular source of CXCL9 and CXCL10 in the testis during infection-induced orchitis.

### Lactylation promotes *Cxcl9* and *Cxcl10* expression in macrophages at the transcriptional level

**Figure 3:**
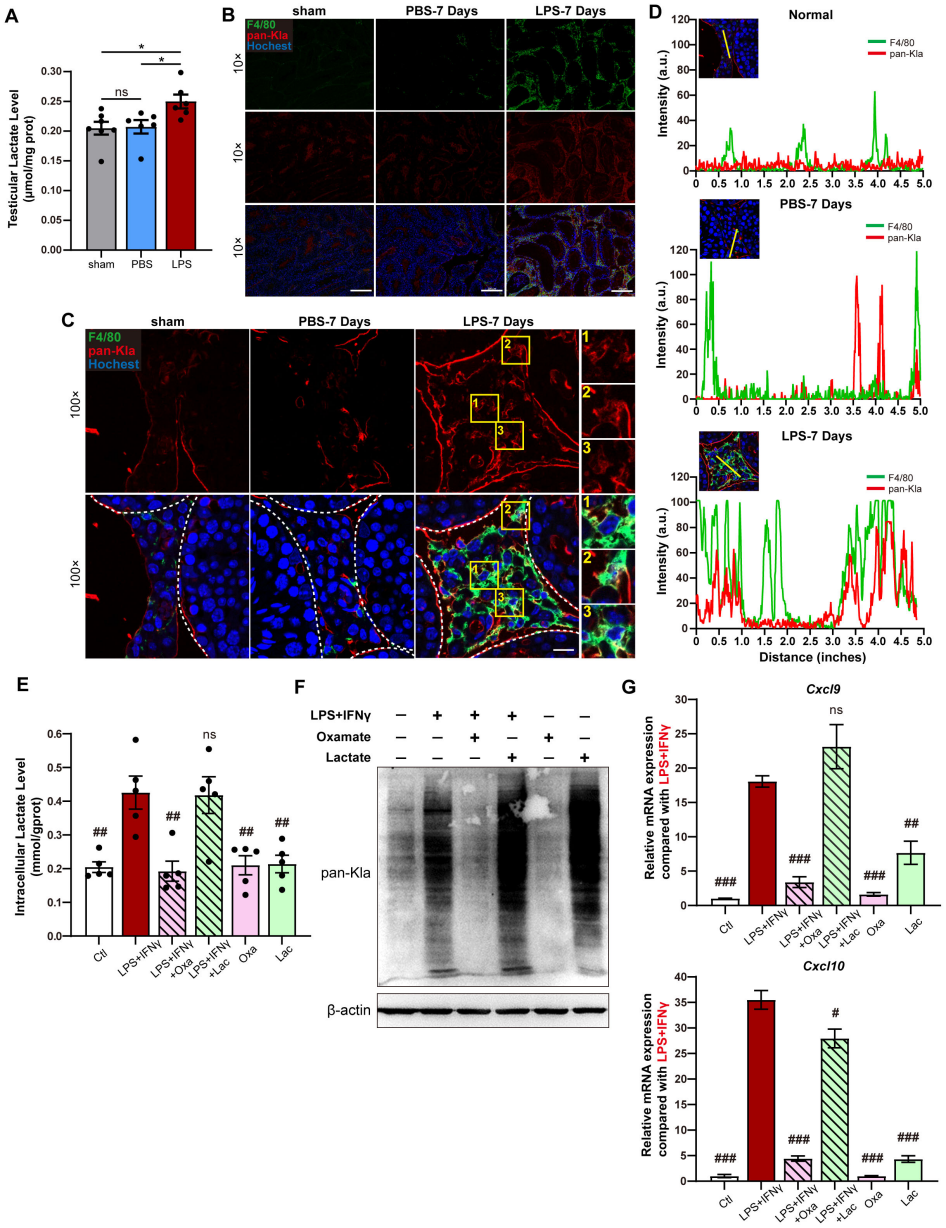
Lactylation promotes the transcriptional expression of CXCL9 and CXCL10 in macrophages. **(A)** The lactate levels in the testes from LPS-induced orchitic mice, PBS control, and sham group (*n* = 6). **(B)** Representative immunofluorescence images showing the general localization of lysine lactylation modification (pan-Kla) and F4/80^+^ macrophages in the testes from mice mentioned in **(A)**. Scale bar, 200 μm at 10× magnification. **(C)** More precise co-localization of pan-Kla-positive signals and F4/80^+^ macrophages in the testicular interstitium under an oil immersion microscope. White dotted lines indicate the edge of the seminiferous tubules, and three yellow boxes indicate typical F4/80^+^ macrophages in the testicular interstitium. Scale bar, 20 μm at 100× magnification. **(D)** Co-localization analysis of pan-Kla and F4/80 fluorescent signals in the testes from LPS-induced orchitic mice, PBS control, or sham group. **(E)** Intracellular lactate levels of RAW264.7 cells 24 h after LPS + IFN-γ stimulation with/without oxamate (Oxa) or lactate (Lac) treatment, or after incubating with Oxa or Lac alone. RAW264.7 cells without LPS + IFN-γ stimulation served as control (Ctl). Pound signs (#) indicate comparison with the LPS + IFN-γ stimulation group (*n* = 5, normalized with intracellular protein levels). **(F)** Global lysine lactylation levels in RAW264.7 cells from the groups mentioned in **(E)**. **(G)** Relative mRNA levels of *Cxcl9* and *Cxcl10* in RAW264.7 cells from the groups mentioned in **(E)** (*n* = 3). Pound signs (#) indicate comparison with the LPS + IFN-γ stimulation group. Data are statistically compared with one-way ANOVA. Error bars represent mean ± SEM. *p < 0.05, ##*p* < 0.01, ###*p* < 0.001; ns, not significant.

The level of lactate was significantly increased in the testis during LPS-induced orchitis (**Figure 3A**). Because of the pivotal role of lactylation in lactate function (14), we hypothesized that lactylation may be involved in the modulation of macrophage function in the testis during infection-induced orchitis. Immunofluorescence analysis showed that the global lysine lactylation (pan-Kla) was substantially increased in testicular interstitial tissue in mice with LPS-induced orchitis (**Figure 3B**), which was co-localized with macrophages (**Figures 3C, D**), indicating an increase in the lactylation in macrophages during LPS-induced orchitis. Given that interferon-γ (IFN-γ) is an abundant cytokine and plays a key role in macrophage function during infection-induced orchitis (21), we stimulated macrophages with LPS and IFN-γ to investigate whether and how lysine lactylation was involved in the regulation of *Cxcl9* and *Cxcl10* mRNA expression in macrophages. As expected, lactate production and subsequent lactylation were considerably increased in a time-dependent manner in macrophages treated with LPS and IFN-γ (**Supplementary Figures 6A, B**), accompanied by increased mRNA levels of *Cxcl9* and *Cxcl10* (**Supplementary Figure 6C**). Sodium oxamate (Oxa) is widely used as an inhibitor of lactylation due to inhibition of lactic dehydrogenase (LDH) activation and intracellular lactate production without affecting other processes in glucose metabolism (14, 22, 23), while lactate is viewed as an activator of lactylation (**Supplementary Figure 6D**). Indeed, the inhibition of lactylation by oxamate reduced the levels of intracellular lactate (**Figure 3E**) and lactylation (**Figure 3F**) and, as a consequence, abolished the increase in the *Cxcl9* and *Cxcl10* transcripts in macrophages treated with LPS and IFN-γ (**Figure 3G**). Taken together, these results highlight lactylation-mediated transcriptional control of *Cxcl9* and *Cxcl10* in macrophages during infection-induced orchitis.

### Lactylation promotes CXCL9 and CXCL10 transcription in macrophages by inhibiting the ubiquitin–proteasome pathway-mediated STAT1 degradation

**Figure 4:**
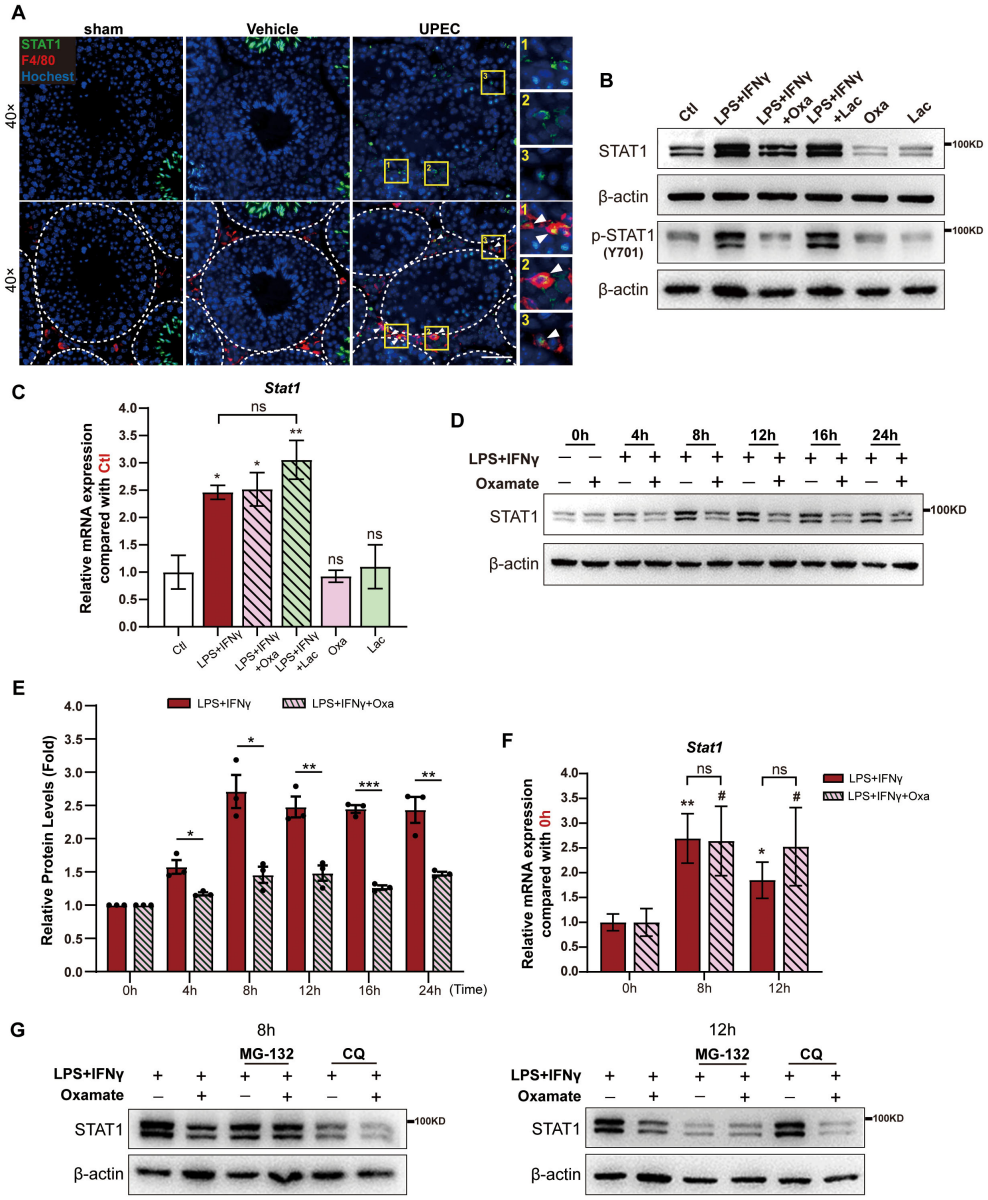
Lactylation in macrophages inhibited ubiquitin–proteasome pathway-mediated STAT1 degradation. **(A)** Representative immunofluorescence images showing the co-localization of STAT1 and F4/80^+^ macrophages in the testicular interstitium from UPEC-induced orchitic mice, vehicle control, or sham group. Scale bars, 50 μm at 40× magnification. **(B)** Protein levels of STAT1 and p-STAT1 (tyrosine 701 phosphorylated) in RAW264.7 cells 24 h after LPS + IFN-γ stimulation with/without oxamate (Oxa) or lactate (Lac) treatment, or after incubating with Oxa or Lac alone. RAW264.7 cells without LPS + IFN-γ stimulation served as control (Ctl). **(C)** Relative mRNA levels of *Stat1* in RAW264.7 cells in the groups mentioned in **(B)**. Asterisks (*) indicate comparison with the Ctl group (*n* = 3). **(D)** Time course of intracellular STAT1 level in RAW264.7 cells over 24 h after LPS + IFN-γ stimulation, with or without Oxa treatment, measured at 4-h intervals. **(E)** The gray value analysis of STAT1 protein bands in **(D)** (*n* = 3). The value is normalized with the gray value of β-actin and the fold is normalized with 0 (h) **(F)** Relative mRNA levels of *Stat1* in RAW264.7 cells at 8 and 12 h after LPS + IFN-γ stimulation, with or without Oxa treatment (*n* = 3). Asterisks (*) indicate comparison with the LPS + IFN-γ-treated group at 0 h, and pound signs (#) indicate comparison with the LPS + IFN-γ + Oxa-treated group at 0 (h) **(G)** Intracellular STAT1 level in RAW264.7 cells stimulated with LPS + IFN-γ or LPS + IFN-γ + Oxa, with or without MG-132 or chloroquine treatment for 8 or 12 (h) Two groups of data are statistically compared with Student’s *t-*test, while multiple groups of data are statistically compared with one-way ANOVA. Error bars represent mean ± SEM. *#*p* < 0.05, **p < 0.01, ***p < 0.001; ns, not significant.

Next, we further investigated the molecular mechanisms underlying lactylation-mediated transcriptional control of *Cxcl9* and *Cxcl10* in macrophages. Signal transducer and activator of transcription 1 (STAT1), a master transcription factor of *Cxcl9* and *Cxcl10* (24, 25), was significantly increased in intratesticular macrophages during UPEC-induced orchitis (**Figure 4A**). Interestingly, inhibition of lactylation by oxamate significantly reduced the protein levels of both STAT1 and p-STAT1 in macrophages stimulated with LPS and IFN-γ, without affecting the mRNA levels of *Stat1* at any time points (**Figures 4B–F**). These results raise the possibility that lactylation-mediated post-transcriptional control of STAT1 increases the mRNA level of Cxcl9 and *Cxcl10* in macrophages. The ubiquitin–proteasome pathway and the autophagy–lysosome pathway are the most important pathways for post-transcriptional control of the protein level (26). Of note, inhibition of lactylation failed to reduce the protein level of STAT1 in macrophages treated with LPS and IFN-γ when inhibiting the ubiquitin–proteasome pathway by using MG-132 (**Figure 4G**). Meanwhile, inhibition of the autophagy–lysosome pathway by chloroquine (CQ) had no demonstrable effect on lactylation inhibition-mediated reduction of STAT1 protein level in macrophages treated with LPS and IFN-γ (**Figure 4G**). Taken together, these results demonstrate that lactylation significantly upregulates the transcript levels of *Cxcl9* and *Cxcl10* in macrophages by inhibiting ubiquitin–proteasome pathway-mediated STAT1 degradation.

### TRIM21 lactylation inhibits STAT1 degradation by constraining the binding of TRIM21 to STAT1

**Figure 5:**
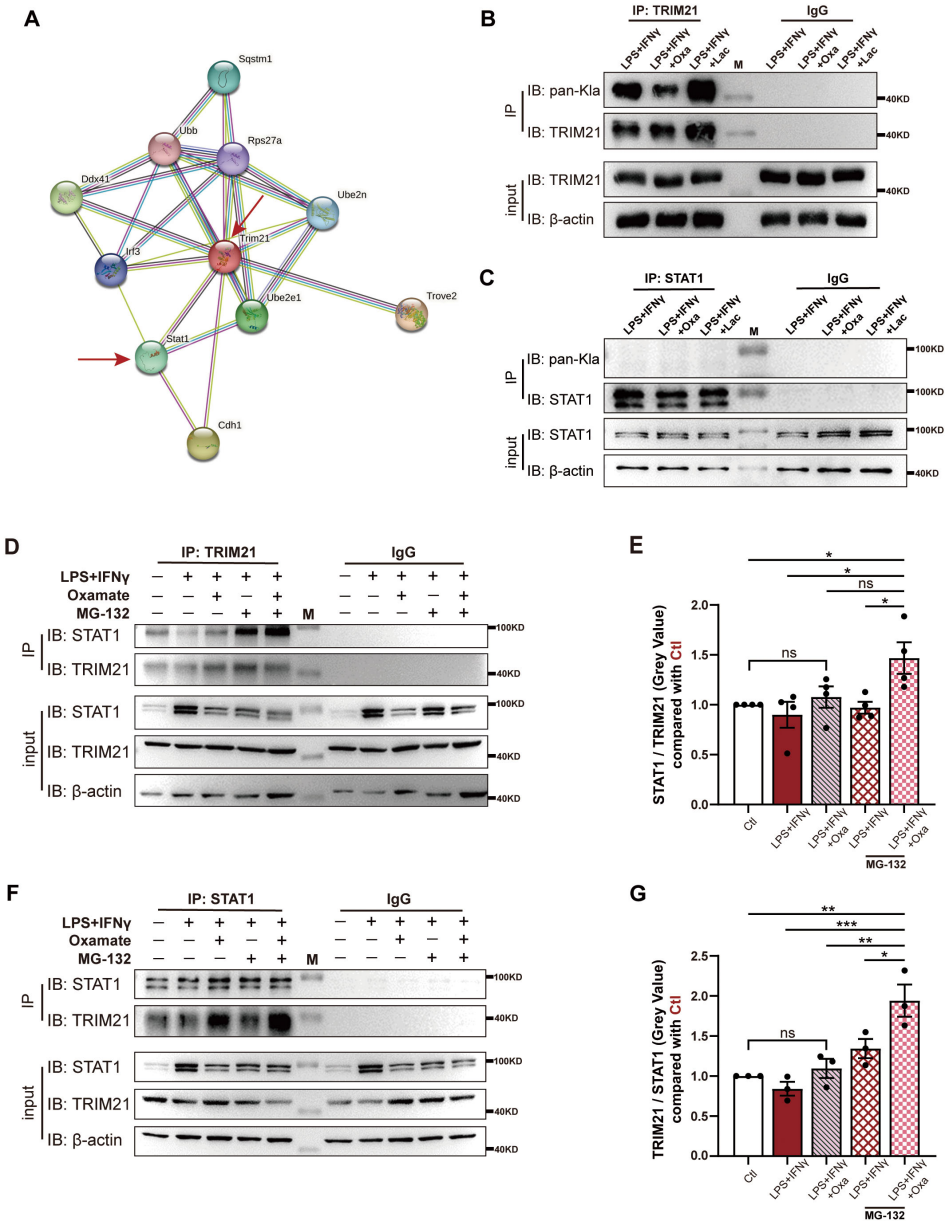
TRIM21 lactylation inhibits STAT1 degradation by constraining the binding of TRIM21 to STAT1. **(A)** Predicted interaction between TRIM21 and STAT1 based on the STRING database. The interaction network is centered on TRIM21, with another red arrow indicating STAT1. Most TRIM21-interacting proteins are involved in ubiquitination. **(B)** Proteins from RAW264.7 cells stimulated with LPS + IFN-γ/LPS + IFN-γ + Oxa/LPS + IFN-γ + Lac were pulled down by the TRIM21 antibody and detected with the anti-lactyllysine antibody. LPS + IFNγ + Lac-treated RAW264.7 cells served as the positive control. **(C)** Proteins from RAW264.7 cells stimulated with LPS + IFN-γ/LPS + IFN-γ + Oxa/LPS + IFN-γ + Lac were pulled down by the STAT1 antibody and detected with the anti-lactyllysine antibody. **(D)** Proteins from RAW264.7 cells incubated with LPS + IFN-γ/LPS + IFN-γ + Oxa/LPS + IFN-γ + MG-132/LPS + IFN-γ + Oxa + MG-132 were pulled down by the TRIM21 antibody and detected with the anti-STAT1 antibody. RAW264.7 cells without any treatment served as the negative control. M, marker, the same below. **(E)** Statistics of gray value analysis in **(E)** (*n* = 4, normalized with the gray value of β-actin). **(F)** Proteins from RAW264.7 cells in the groups mentioned in **(D)** were pulled down by the STAT1 antibody and detected with the anti-TRIM21 antibody. **(G)** Statistics of gray value analysis in **(F)** (*n* = 3, normalized with the gray value of β-actin). Data are statistically compared with one-way ANOVA. Error bars represent mean ± SEM. *p < 0.05, **p < 0.01, ***p < 0.001; ns, not significant.

Tripartite-motif protein 21 (TRIM21), an important E3 ubiquitin ligase, plays a key role in the regulation of the ubiquitin–proteasome pathway by directly binding the target protein and subsequently catalyzing the ubiquitination process (27–29). Given that non-histone lactylation can affect the binding of lactylation-modified protein to other proteins (30), we wonder whether lactylation inhibits STAT1 degradation by constraining the binding of TRIM21 to STAT1. The STRING database analysis showed a relatively clear interplay between TRIM21 and STAT1 (**Figure 5A**). Meanwhile, TRIM21 lactylation was strongly increased in macrophages treated with LPS and IFN-γ (**Figure 5B**), while STAT1 lactylation was not observed in macrophages (**Figure 5C**). Inhibition of lactylation by oxamate markedly reduced TRIM21 lactylation in macrophages (**Figure 5B**). More importantly, inhibition of TRIM21 lactylation markedly increased the binding of TRIM21 to STAT1 in the presence of MG-132, concomitant with reduced protein levels of STAT1 in the absence of MG-132 (**Figures 5D–G**). In these experiments, MG-132 was employed to prevent the degradation of STAT1 after binding for facilitating the detection of protein–protein interaction. Taken together, these results indicate that TRIM21 lactylation inhibits STAT1 degradation by constraining the binding of TRIM21 to STAT1 in macrophages.

### TRIM21 K345 lactylation impairs the binding of TRIM21 to STAT1

**Figure 6:**
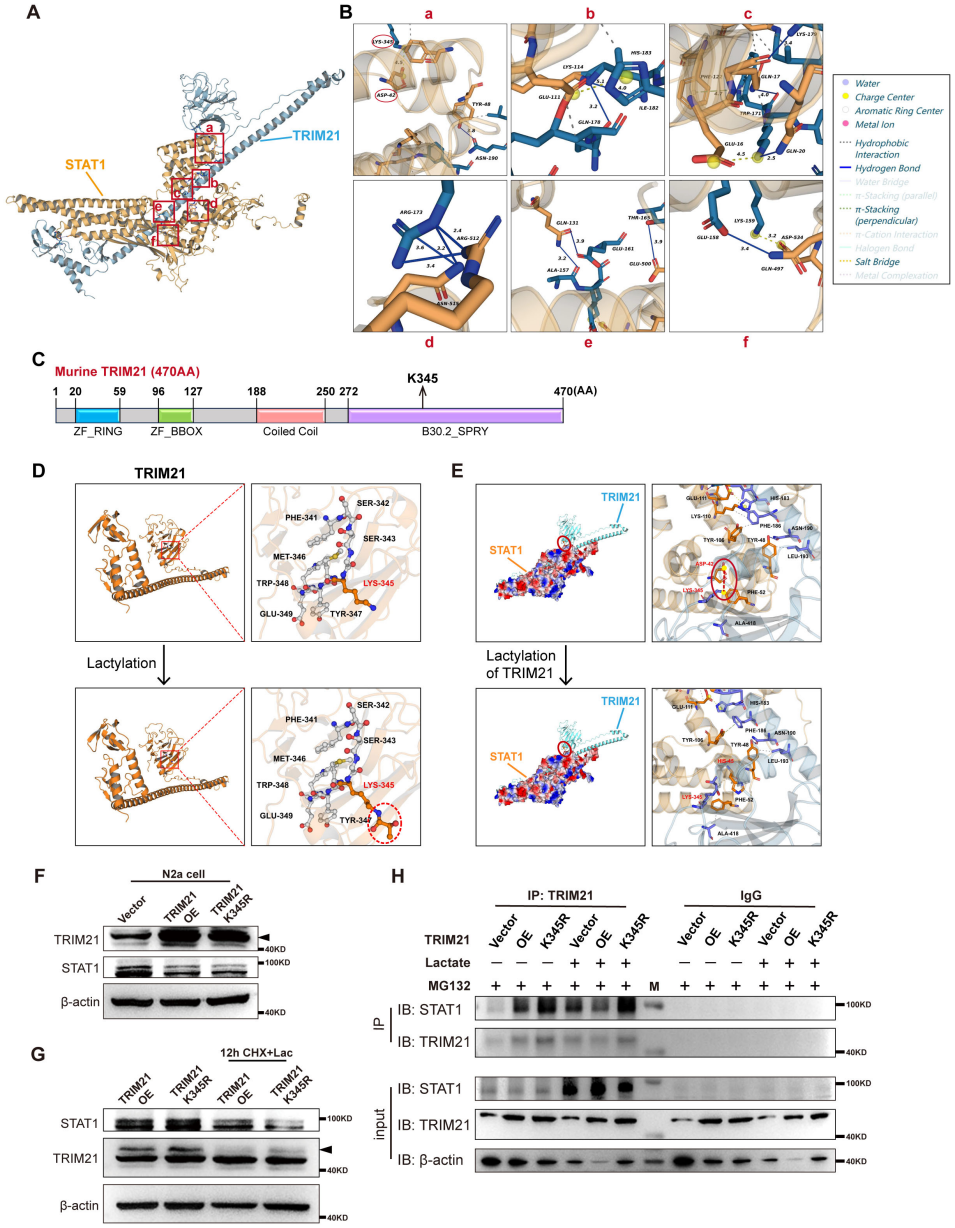
TRIM21 K345 lactylation impairs the binding of TRIM21 to STAT1. **(A)** The interaction model between TRIM21 and STAT1 predicted by the HDOCK server (docking score = −219.68, confidence score = 0.8012). The red boxes highlight six interaction sites between these two proteins. **(B)** Detailed interactions between amino acids in six interaction sites between TRIM21 and STAT1. **(C)** Schematic diagram showing four functional domains on murine TRIM21, with their respective start and end amino acid positions. **(D)** The structure of TRIM21 before and after the lactylation modification at K345 (marked by a red dashed circle). **(E)** Protein–protein docking pose (interface) of murine TRIM21 and STAT1 before and after K345 lactylation of TRIM21. **(F)** Intracellular TRIM21 and STAT1 levels in N2a cells after transfection of empty vector, TRIM21, and TRIM21 K345R overexpression plasmids for 24 (h) The black arrow indicates the major TRIM21 protein band (the same below). OE, overexpression. **(G)** Intracellular TRIM21 and STAT1 levels in N2a cells transfected with TRIM21 and TRIM21 K345R overexpression plasmids and treated with cycloheximide (CHX) and 10 mM of lactate for 12 (h) **(H)** Cell proteins of N2a cells in the three groups mentioned above, treated with or without lactate, were pulled down by the TRIM21 antibody and detected with the anti-STAT1 antibody.

To investigate the molecular basis of how TRIM21 lactylation impairs the binding of TRIM21 to STAT1, we predicted the interaction model of TRIM21 and STAT1 with the HDOCK server by sampling six degrees of freedom (three translational and three rotational) (**Figure 6A**). We next analyzed the six potential interaction sites between TRIM21 and STAT1 in this model and found three sites on TRIM21 containing lysine residues, namely, K345 (a), K179 (c), and K159 (f) (**Figures 6A, B**). However, only K345 is located on the domain with protein-binding activity (SPRY domain) (31) among these three lysine residues (**Figure 6C**). We used three-dimensional modeling to predict the structure of TRIM21 with or without K345 lactylation modification and found that the conformation of the lysine residue would change apparently following K345 lactylation modification of TRIM21 (**Figure 6D**), leading to the break of the salt bridge between TRIM21 K345 and STAT1 D42 (**Figure 6E**). We further tested whether K345 mutation in TRIM21 could rescue the lactylation-mediated impairment of the interaction between TRIM21 and STAT1. K345 mutation in TRIM21 had no demonstrable effect on the degradation of STAT1 in N2a cells in the absence of lactate, as shown by the comparable levels of STAT1 in N2a cells transfected with WT TRIM21 or TRIM21 K345R (**Figure 6F**). However, K345 mutation in TRIM21 increased the degradation of STAT1 in N2a cells in the presence of lactate (**Figure 6G**), accompanied by an increased binding of TRIM21 to STAT1 (**Figure 6H**). Taken together, these results suggested that TRIM21 K345 lactylation impairs the binding of TRIM21 to STAT1, inhibits ubiquitin–proteasome pathway-mediated STAT1 degradation, and as a consequence, promotes the transcriptional expression of CXCL9 and CXCL10 in macrophages during infection-induced orchitis.

## Discussion

Infection-induced orchitis is one of the major causes of acquired male infertility, but the mechanisms underlying this process are poorly understood. In this study, we demonstrated that macrophage TRIM21 lactylation promoted the mRNA expression of *Cxcl9* and *Cxcl10* in a STAT1-dependent manner to recruit inflammatory cells into the testis and, as a consequence, impair spermatogenesis in seminiferous tubules during infection-induced orchitis. Furthermore, the K345 lactylation of TRIM21 inhibited ubiquitin–proteasome pathway-mediated STAT1 degradation and consequently enhanced the expression of *Cxcl9* and *Cxcl10* in macrophages by restraining the binding of TRIM21 to STAT1. Our findings suggest that the non-histone protein TRIM21 lactylation is involved in the regulation of macrophage-associated inflammation, thus revealing possible therapeutic targets for the treatment of infection-induced orchitis.

During testicular inflammation, many chemokines were induced; however, our flow cytometric data showed that macrophages, T cells, and dendritic cells were the predominant infiltrating leukocytes, suggesting active recruitment from the circulation rather than local expansion. We therefore focused on chemokine pathways that can concomitantly attract these populations. The IFN-inducible CXCR3 ligands CXCL9, CXCL10, and CXCL11 are prototypical Th1-type chemokines that guide activated CD4^+^ and CD8^+^ T cells, macrophages, and myeloid cells into inflamed tissues and are central regulators of leukocyte trafficking (32). In the testis, CXCL10 is robustly induced in Sertoli cells, Leydig cells, and testicular macrophages in response to viral or inflammatory stimuli, where CXCL10–CXCR3 signaling promotes leukocyte infiltration, impairs steroidogenesis, and aggravates germ cell damage (33). Guided by this biology, we initially examined *Cxcl9*, *Cxcl10*, and *Cxcl11* but found that CXCL9 and CXCL10 showed the strongest and most reproducible induction at both the mRNA and protein levels in our model, whereas *Cxcl11* expression was low and its protein barely detectable, consistent with the generally weaker *Cxcl11* expression reported in mice (34). We therefore concentrated our mechanistic analyses on CXCL9 and CXCL10 as the dominant and representative CXCR3 ligands in testicular inflammation while acknowledging that CXCL11 belongs to the same axis and that other chemokine pathways may also act in parallel.

Macrophages were identified as the majority of infiltrated inflammatory cells in the testis and contributed to spermatogenesis impairment in mice during infection-induced orchitis. Consistent with these observations, the testicular biopsy samples with orchitis caused by pathogens, such as UPEC, *Francisella*, and *Salmonella* Typhi, show impaired seminiferous tubules and reduced sperm in the seminiferous tubule lumen, accompanied by increased macrophage infiltration in the testicular interstitium (3, 35, 36). The combined blockade of CXCL9 and CXCL10 plays an optimal role in ameliorating infection-induced orchitis. This finding, in conjunction with a previously published study showing that CXCL10 deficiency only partially alleviated LPS-induced testicular damage (37), suggests that macrophages exacerbate infection-induced orchitis by producing CXCL9 and CXCL10.

In physiological conditions, testicular macrophages are not only the most abundant leukocytes but also exhibit an M2-like, IL-10-producing phenotype that is essential for maintaining immune privilege and restraining leukocyte recruitment in the testis (38). Thus, depleting these immunoregulatory cells is expected to remove a key brake on inflammation rather than simply “subtract” CD45^+^ cells. Consistent with the reports involving other organs, clodronate liposome-mediated macrophage depletion can induce local inflammatory signals and paradoxically enhance neutrophil and monocyte influx (39). In the testis, experimental models inducing macrophage loss similarly show increased small mononuclear cells and granulocytes in the interstitium (40). In this context, the three- to fourfold rise in total CD45^+^ cells that we observed after clodronate liposome treatment (**Figure 2B**) is most likely explained by secondary recruitment of non-macrophage leukocytes into a microenvironment where macrophage-mediated immune regulation has been disrupted, rather than by a technical artifact.

Lactate plays a pleiotropic role in the regulation of macrophage function, but the exact molecular basis remains elusive. For instance, lactate promotes the expression of *Tnf-a* in heparin-binding protein-treated macrophages by the activation of the NF-κB pathway (41) and enhances IL-1β production in adipose macrophages by the stabilization of HIF-1α (11). Consistent with the published literature, we found that lactate induced the increased mRNA expression of *Cxcl9* and *Cxcl10* in macrophages during infection-induced orchitis in a STAT1-dependent manner. Lactylation, a post-translational modification, is a pivotal component of lactate function and is involved in multiple physiological and pathological processes (42). Accumulating evidence supports that histone lactylation is involved in the regulation of macrophage function, but it promotes the switch from an inflammatory phenotype to a steady-state phenotype in macrophages (43). We demonstrated that lactate promoted CXCL9 and CXCL10 production in inflammatory macrophages, raising the possibility that non-histone lactylation may contribute to lactate-mediated modulation of inflammatory macrophage functions. Consistent with this notion, we found that TRIM21 lactylation promoted the increase of *Cxcl9* and *Cxcl10* transcripts in macrophages in a STAT1-dependent manner during infection-induced orchitis.

TRIM21, an E3 ubiquitin ligase, interacts with the target protein, transfers the ubiquitin molecule to the target protein, and promotes the ubiquitination-mediated degradation of the target protein (44). Indeed, lysine lactylation antibody immunoprecipitation and mass spectrometry analysis indicate that TRIM21 could be modified by lactylation in brain cells (45). Furthermore, non-histone lactylation can modulate the interactions of lactylation-modified protein with other proteins. For instance, non-histone MOESIN Lys72 lactylation enhances the interaction with transforming growth factor β receptor I in Treg cells (46). In addition, non-histone PKM2 K62 lactylation inhibits its tetramer-to-dimer transition in macrophages (47). In line with these published reports, our findings showed that TRIM21 K345 lactylation impairs the interaction with STAT1 and, as a consequence, inhibits the ubiquitination-mediated degradation of STAT1.

Evidence from other systems and diseases suggests that histone acetyltransferases such as p300/CBP may function as potential “writers” of lysine lactylation (48, 49), while histone deacetylases (HDACs) (23) and Sirtuin (SIRT) family proteins (50) may act as “erasers” of lysine lactylation. In round/elongating spermatids, p300 and/or CBP enhance acetylation of histone H4, which is essential for the completion of histone-to-protamine exchange (51). SIRT1 and SIRT3 show intrinsic direct activity in developing male germ cells during spermatogenesis and cooperate to protect germ cells from oxidative stress (52). However, the functions of p300/CBP and SIRT in testicular macrophages under physiological or inflammatory conditions have not been reported. In testicular macrophages, a high-dose LPS treatment (5.0 mg/kg) markedly downregulated HDAC5 in 8 h, leading to the M1 polarization of testicular macrophages (53). Whether this process is related to the HDAC-mediated erasure of lysine lactylation remains to be further investigated. According to a mouse RNA-seq dataset offered by Rukmali et al. (54), the lysine-lactylation “writers” EP300 (p300) and KAT8 (17) show detectable transcripts in the testis. Meanwhile, the lactylation “erasers” (HDAC1–3, HDAC5, SIRT1-3) are also expressed in the testis according to this RNA-seq consensus. This indicates that certain lactylation and delactylation enzymes are constitutively expressed in the testis, supporting the biological plausibility of lactylation dynamics in orchitis. In addition, we noted that transcript presence does not equate to enzymatic activity, and the precise expression patterns of these enzymes in the human testis remain to be further explored. Future studies will be required to delineate the exact enzymes and regulatory pathways mediating TRIM21 lactylation in testicular macrophages.

Although RAW264.7 macrophages were used as an *in vitro* model in this study, they cannot fully represent the unique features of resident or peripherally derived macrophages in the testis. Therefore, our findings regarding TRIM21 lactylation and chemokine CXCL9 and CXCL10 production should be interpreted with caution, and future studies using primary testicular macrophages and *in vivo* models will be necessary to validate these results.

In summary, TRIM21 K345 lactylation increases the mRNA expression of *Cxcl9* and *Cxcl10* in macrophages by inhibiting ubiquitination-mediated STAT1 degradation, which results in the infiltration of inflammatory cells and, as a consequence, impairs spermatogenesis in mice during infection-induced orchitis. Our findings identify that excessive *Cxcl9* and *Cxcl10* expression drives pathological inflammation and testicular damage in infectious orchitis, and TRIM21 lactylation acts as an upstream regulatory mechanism promoting chemokine expression in macrophages. Moreover, targeting TRIM21 lactylation (e.g., inhibiting LDH, inhibiting lactylation enzymes’ activity) or neutralizing CXCL9/CXCL10 may offer a promising adjuvant therapeutic strategy to preserve testicular function, pending further validation in preclinical models.

## Methods

### Mice

Our study exclusively examined male mice because the disease modeled is only relevant in males. Eight-week-old C57BL/6J mice were purchased from the Animal Core Facility of Nanjing Medical University (Nanjing, China). All mice were housed under controlled environmental conditions (20°C–22°C with a 12-h light/dark cycle, relative humidity between 40% and 60%, and under specific pathogen-free conditions). All the animal experiments were performed in compliance with the Ethical Procedures for Experimental Animal Welfare and approved by the Institutional Animal Care and Use Committee (IACUC) of Nanjing Medical University (Approval Number: IACUC-2109046).

### Bacteria culture and animal treatment

The uropathogenic *E. coli* (UPEC) strain, CFT073, was kindly provided by Professor Shuo Yang from Nanjing Medical University. UPEC was cultured overnight at 37°C on LB plates (without antibiotics) after gradient dilution, and colonies were counted the next day. After colony counting, a single clone of UPEC was isolated and inoculated in lysogeny broth (LB) medium at 37°C until it grew to an exponential phase (OD_600_ = 0.5–1.0). Then, the medium was centrifuged at 4,500×*g* for 10 min at 4°C, and the pellets were washed twice with cold phosphate-buffered saline (PBS). UPEC was stored in Dulbecco’s modified Eagle’s medium (DMEM) at 4°C for 24 h.

Orchitis was induced by UPEC or LPS (ST1470, Beyotime, Shanghai, China) in male mice as described previously with some modifications (18, 20). Mice were anesthetized by injecting pentobarbital intraperitoneally (50 mg/kg; P3636, Sigma-Aldrich, Burlington, VT, USA), and the testes and vas deferens were exposed through a small incision in the scrotum. Then, 1 × 10^5^ colony-forming units (CFU) of UPEC in 10 μL of DMEM, 10 μL of DMEM as control, 30 μg of LPS in 10 μL of PBS, or 10 μL of PBS as control was injected into the upper pole of the testis with a 25-µL microsyringe (702N, Hamilton, Bonaduz, Switzerland) fitted with a 33-gauge needle. The injection was performed slowly and at a uniform rate under a stereomicroscope, avoiding the rete testis located at the posterior margin of the testis. Mice of the sham group only received anesthesia and surgery. The vas deferens of the UPEC group was ligated with surgical sutures to prevent the spread of the infection. After the wound was sutured, the mice were kept warm until they recovered. Their wounds were disinfected with iodophor daily. The time point was chosen based on the data acquired in previous research (55). On the 7th day after inducing orchitis, pronounced histological damage and immune cell infiltration were observed in both the testis and epididymis.

### *In situ* chemokine neutralization

The recombinant neutralizing antibodies anti-CXCL9 (AF-492-NA, R&D Systems, Minneapolis, MN, USA) and anti-CXCL10 (AF-466-NA, R&D Systems, Minneapolis, MN, USA) were diluted in sterile PBS and stored at −20°C. Adult male C57BL/6J mice were anesthetized, and the testes were exposed via scrotal incision as mentioned above. For *in situ* neutralization, 10 µg of anti-CXCL9 and/or 10 µg of anti-CXCL10 and 30 µg of LPS (all in 10 μL of solution) were premixed and delivered into the testis using a microsyringe. The control group received the same amount of LPS and isotype IgG (AB-108-C, R&D Systems, Minneapolis, MN, USA) injection. Following incision closure, mice were allowed to recover (day 0). The same procedure was repeated on days 3 and 6 with antibody injections only (without LPS).

### Testicular macrophage depletion

Clodronate liposome (40337ES08, Yeasen, Shanghai, China) or control (PBS) liposome (40338ES05, Yeasen, Shanghai, China) was prepared as 200 µL aliquots and gently warmed to room temperature before use. Adult male mice were anesthetized and the testes were injected *in situ* with 10 µL of clodronate liposomes (5 µg/µL) or equal PBS liposomes using a microsyringe. After closure of the incision, mice were allowed to recover, and the testes were collected 3 days later to assess macrophage depletion or for subsequent orchitis induction.

### Cell culture

Murine RAW264.7 macrophages were purchased from the ATCC and cultured in DMEM (11965092, Gibco, Grand Island, NY, USA) supplemented with penicillin–streptomycin (100 U/mL; 15140122, Gibco, Grand Island, NY, USA) and 10% fetal bovine serum (FBS; 16000-044, Gibco, Grand Island, NY, USA). To induce lysine lactylation, macrophages were treated with 10 mM of L-lactic acid (A419983, Sangon Biotech, Shanghai, China) for 30 min followed by LPS (100 ng/mL) and IFN-γ (20 ng/mL; 39127S, CST, Danvers, MA, USA) stimulation. To inhibit lactate production, macrophages were treated with 20 mM of sodium oxamate (A600871, Sangon Biotech, Shanghai, China) for 2 h, followed by LPS and IFN-γ stimulation.

### Histological analysis

The isolated testes and epididymis were fixed in modified Davidson’s fixative solution (MDF; 1.2% formaldehyde, 15% ethyl alcohol, and 5% glacial acetic acid) at room temperature for 48 h. The testes needed to be cut in half after being fixed for 24 h. Subsequently, tissues were dehydrated in a series of ethanol concentrations (70%, 80%, 90%, and 100%) and immersed in a solution of xylene and ethanol (1:1) and then in 100% xylene. These tissues were embedded in paraffin, cut into 5-μm-thick sections, mounted on slides, deparaffinized, rehydrated, and stained with hematoxylin (G1004, Servicebio, Wuhan, China) and eosin (E607321, Sangon Biotech, Shanghai, China).

For histological analysis of the testis, the diameter of seminiferous tubules and the thickness of seminiferous epithelium were measured using ImagePro Plus software (v.6.0; Media Cybernetics, USA) from 20 tubules (long axis:short axis < 1.2:1) at ×20 magnification of each mouse, according to the methods in a previous study (56). Briefly, the diameter was calculated as the mean of the long and short axes of the seminiferous tubules. We measured the thinnest part of the seminiferous epithelium as its thickness.

### Epididymal sperm analysis

The epididymis was stripped out from the fat pad; then, the cauda epididymis was dissected and suspended in modified HTF medium (90126, Irvine Scientific, Tokyo, Japan) and supplemented with 10% FBS (16000-044, Gibco, Grand Island, NY, USA), at 37°C for 5 min. Subsequently, 10 μL of the sperm suspension was assessed for motility characteristics at 37°C by computer-assisted semen analysis (CASA; Hamilton Thorne Research Inc., USA). At least six fields of view were selected for each sample. Specifically, we measured the motility and progressive motility. Sperm count was evaluated with a hemocytometer. The sperm cells were spread on glass slides and fixed in 4% paraformaldehyde (P6148, Sigma-Aldrich, Burlington, VT, USA) at room temperature for 40 min, after which H&E staining and morphological observation were performed.

### Total RNA isolation and quantitative real-time PCR

Tissues and cells were homogenized in TRIzol™ reagent (15596026, Invitrogen, Waltham, MA, USA), and RNA was extracted as described by the manufacturer. Total RNA (1 μg) was reverse-transcribed with 5× All-In-One MasterMix (G592, Applied Biological Materials, Vancouver, Canada). Then, the products were amplified with BlasTaq Green 2× qPCR MasterMix (G895, Applied Biological Materials, Vancouver, Canada) on the Q5 Real-Time PCR System (Applied Biosystems, USA). The relative expression levels of mRNAs were assessed using the 2^−ΔΔCt^ method, and normalization was performed with 18S rRNA or β-actin primers. The specificity of the PCR products was assessed by melting curve analyses. All primer sequences are listed below.

**Table:** 

	F (5′→3′)	R (5′→3′)
*Il-1β*	5′-GATCCACACTCTCCAGCTGCA-3′	5′-CAACCAACAAGTGATATTCTCCATG-3′
*Il-6*	5′-CCAAGAGGTGAGTGCTTCCC-3′	5′-CTGTTGTTCAGACTCTCTCCCT-3′
*Tnf-a*	5′-CCCTCACACTCAGATCATCTTCT-3′	5′-GCTACGACGTGGGCTACAG-3′
*Nos2*	5′-GCCACCAACAATGGCAACA-3′	5′-CGTACCGGATGAGCTGTGAATT-3′
*Il-10*	5′-ACTTTAAGGGTTACTTGGGTTGC-3′	5′-ATTTTCACAGGGGAGAAATCG-3′
*Cxcl9*	5′-TCTCGGACTTCACTCCAACACA-3′	5′-ACTCCACACTGCTGGAGGAAGA-3′
*Cxcl10*	5′-CCGTCATTTTCTGCCTCATCC-3′	5′-CCCTATGGCCCTCATTCTCA-3′
*Cxcl11*	5′-GAACAGGAAGGTCACAGCCATAGC-3′	5′-TCAACTTTGTCGCAGCCGTTACTC-3′
*Ccl17*	5′-AGTGCTGCCTGGATTACTTCAAAG-3′	5′-CTGGACAGTCAGAAACACGATGG-3′
*Ccl22*	5′-TAACATCATGGCTACCCTGCG-3′	5′-TGTCTTCCACATTGGCACCA-3′
*Stat1*	5′-GTTCCGACACCTGCAACTGAA-3′	5′-GAGGTGGTCTGAAAGGGAACAA-3′
*18S*	5′-CGGACAGGATTGACAGATTGATAG-3′	5′-ATGCCAGAGTCTCGTTCGTTAT-3′
*β-actin*	5′-GGCTGTATTCCCCTCCATCG-3′	5′-CCAGTTGGTAACAATGCCATGT-3′

### Protein extraction and Western blotting analysis

Proteins were extracted using 8 mol/L of urea buffer (composed of 50 mmol/L of Tris–HCl pH 8.2, 75 mmol/L of NaCl, and 8 mol/L of urea) supplemented with a protease inhibitor cocktail (B14001, Bimake, Houston, TX, USA), followed by incubation on a shaker at 4°C for 1 h. The lysates were centrifuged at 12,000×*g* for 15 min at 4°C, and then the supernatants were collected to assay protein concentrations with a Bradford protein assay kit (P0006, Beyotime, Shanghai, China). Equal amounts of proteins (15 μg) were separated by electrophoresis on 8% SDS-PAGE gels and then transferred onto polyvinylidene difluoride membranes (1620177, Bio-Rad, USA). After blocking with 5% non-fat milk at room temperature for 2 h, the membranes were incubated with diluted primary antibodies overnight at 4°C. The following primary antibodies were used: anti-β-actin (1:10,000, ABclonal, Wuhan, China, ac026), anti-STAT1 (1:5,000, Proteintech, Wuhan, China, 10144-2-AP), anti-p-STAT1 (1:200, Santa Cruz, Santa Cruz, CA, USA, sc-136229), anti-pan-Kla (1:500, PTM BIO, Hangzhou, China, PTM-1401RM), and anti-TRIM21 (1:5,000, Proteintech, Wuhan, China, 12108-1-AP). Subsequently, the membranes were washed with TBST (Tris-buffered saline with 1‰ Tween-20) and incubated with HRP-conjugated secondary antibodies at room temperature for 2 h. Finally, the signals were visualized through chemiluminescence using ChemiDoc XRS (Bio-Rad).

### Immunoprecipitation

The protein A/G magnetic beads were washed three times with lysis buffer and stored at 4°C. Then, 200 μg of total cellular proteins were incubated with 2 μg of antibodies or isotype control IgG overnight at 4°C on a shaker, followed by adding 60 μL of protein A/G magnetic beads (PB101-02, Vazyme, Nanjing, China) the next day. The beads were washed three times with lysis buffer and boiled in SDS sample buffer. The supernatant was subjected to immunoblotting with appropriate antibodies.

### Immunofluorescence staining and TUNEL assay

Immunofluorescence was performed on dewaxed and rehydrated sections, which were then processed by antigen retrieval using citrate buffer in a high-power microwave oven. Sections were submerged in 5% bovine serum albumin (5% BSA; V900933, VETEC, Burlington, VT, USA) and incubated with the primary antibodies at 4°C overnight. The following primary antibodies were used: anti-SOX9 (1:1,000, Abcam, Cambridge, UK, ab185966), anti-ZO-1 (1:1,000, Proteintech, Wuhan, China, 21773-1-AP), anti-STAT1 (1:1,000, Proteintech, Wuhan, China, 10144-2-AP), anti-CXCL9 (1:1,000, R&D Systems, Minneapolis, MN, USA, AF-492-NA), anti-CXCL10 (1:1,000, R&D Systems, Minneapolis, MN, USA, AF-466-NA), anti-F4/80 (1:500, HUABIO, Hangzhou, China, HA721745), and anti-pan-Kla (1:200, PTM BIO, Hangzhou, China, PTM-1401RM). The sections were washed in PBST (phosphate-buffered saline + 1‰ Tween-20) and incubated with Alexa fluorophore (488 or 555)-conjugated secondary antibodies (Thermo Fischer Scientific, Waltham, MA, USA) at room temperature for 2 h in the dark. The primary/secondary antibodies were diluted in 5% BSA. Nuclear structures were stained with Hoechst 33342 (H3570, Thermo Fisher Scientific, Waltham, MA, USA) at a dilution of 1:1,000 for 10 min, before being covered by glass coverslips and fixed with glycerol. TUNEL assay (A111-03, Vazyme, Nanjing, China) was used to detect testicular cells undergoing apoptosis, following the manufacturer’s protocol. These results were visualized under a laser scanning confocal microscope (LSM800, Carl Zeiss, Germany).

### Lactate measurement

To measure intratesticular or intracellular lactate levels, the tissue or cells were homogenized with an ultrasonic disruptor. Insoluble material was removed by centrifugation at 12,000×*g* for 10 min. The supernatant was collected to measure lactate levels according to the assay kit’s instructions (A019-2-1, Jiancheng Bio, Nanjing, China). The lactate levels were normalized to the protein concentrations of the supernatant.

### Testis digestion and flow cytometry

Testes were prepared as single-cell suspensions as described previously (57). In brief, the testes were decapsulated and digested with 0.5 mg/mL of type IV collagenase (17104019, Gibco, Grand Island, NY, USA), 0.1 mg/mL of DNase I (D4257, Sigma-Aldrich, Burlington, VT, USA), and 2% FBS in RPMI-1640 (11875093, Gibco, Grand Island, NY, USA) at 180 rpm, 37°C for 20 min. Cell suspensions were passed through a 100-μm cell strainer to remove ruptured seminiferous tubules. After being washed twice with cold PBS, the cells were counted using a hemocytometer and blocked with anti-mouse CD16/32 (14-0161-86, Invitrogen, Waltham, MA, USA). Single-cell suspensions were surface-stained with the following antibodies: CD45-Pepey5.5 (eBioscience, San Diego, CA, USA, 45-0451-82, 30-F11), F4/80-APC (BioLegend, San Diego, CA, USA, 123116, BM8), CD11b-PE-Cy7 (BD Pharmingen, Franklin Lakes, NJ, USA, 552850, M1/70), CD11b-FITC (BD Pharmingen, Franklin Lakes, NJ, USA, 554861, OX-42), CD3-V450 (BD Pharmingen, Franklin Lakes, NJ, USA, 560804, 500A2), CD11c-FITC (BioLegend, San Diego, CA, USA, 117305, N418), NK1.1-FITC (eBioscience, San Diego, CA, USA, 11-5941-82, PK136), Ly6G-PE-Cy7 (BioLegend, San Diego, CA, USA, 127617, 1A8), Ly6C-BV421 (BD Pharmingen, Franklin Lakes, NJ, USA, 562727, AL-21), and SiglecF-BV421 (BD Pharmingen, Franklin Lakes, NJ, USA, 562681, E50-2440).

To determine the expression of intracellular chemokines, the cells were stimulated with 25 ng/mL of phorbol 12-myristate 13-acetate (PMA; HY-18739, MCE, Monmouth Junction, NJ, USA), 1 μg/mL of ionomycin (HY-13434, MCE, Monmouth Junction, NJ, USA), and 1 μg/mL of GolgiStop (554724, BD Biosciences, Franklin Lakes, NJ, USA) for 6 h. Then, cells after being surface-stained were fixed and permeabilized with fixation/permeabilization buffer (BD Pharmingen, Franklin Lakes, NJ, USA, 554714) and intracellularly stained with CXCL9-PE (eBioscience, San Diego, CA, USA, 12-3009-80, 2F5.5). For CXCL10 intracellular staining, the primary CXCL10 antibody was purchased from R&D Systems, and Alexa Fluor™ 488 donkey anti-goat IgG antibody (A-11055, eBioscience, San Diego, CA, USA) was used as the secondary antibody. Flow cytometry was performed on FACSVerse (BD Biosciences), and data were analyzed by FlowJo 10.8.1 software.

### Statistical analysis

Statistics and graphing were conducted using GraphPad Prism software (Version 8.0, CA, USA). Comparison of the two groups was analyzed using an unpaired Student’s *t*-test, while multiple comparisons were performed using one-way ANOVA followed by an LSD *post hoc* test. *p*-values are denoted in the figures as follows: *, *p* < 0.05; **, *p* < 0.01; ***, *p* < 0.001; ns, not significant.

## Data Availability

The original contributions presented in the study are included in the article/**Supplementary Material**. Further inquiries can be directed to the corresponding authors.

## References

[1] Eisenberg ML Esteves SC Lamb DJ Hotaling JM Giwercman A Hwang K . Male infertility. Nat Rev Dis Primers. (2023) 9:49. doi: 10.1038/s41572-023-00459-w, PMID: 37709866

[2] Schagdarsurengin U Western P Steger K Meinhardt A . Developmental origins of male subfertility: role of infection, inflammation, and environmental factors. Semin In Immunopathology. (2016) 38:765–81. doi: 10.1007/s00281-016-0576-y, PMID: 27315198

[3] Osegbe DN . Testicular function after unilateral bacterial epididymo-orchitis. Eur Urol. (1991) 19:204–8. doi: 10.1159/000473620, PMID: 1855525

[4] Kristensen DM Desdoits-Lethimonier C Mackey AL Dalgaard MD De Masi F Munkbøl CH . Ibuprofen alters human testicular physiology to produce a state of compensated hypogonadism. Proc Natl Acad Sci United States America. (2018) 115:E715–24. doi: 10.1073/pnas.1715035115, PMID: 29311296 PMC5789927

[5] Ren L Zhang Y Xin Y Chen G Sun X Chen Y . Dysfunction in Sertoli cells participates in glucocorticoid-induced impairment of spermatogenesis. Mol Reprod Dev. (2021) 88:405–15. doi: 10.1002/mrd.23515, PMID: 34032349

[6] Michel V Duan Y Stoschek E Bhushan S Middendorff R Young JM . Uropathogenic Escherichia coli causes fibrotic remodelling of the epididymis. J Pathol. (2016) 240:15–24. doi: 10.1002/path.4748, PMID: 27218225

[7] Wang M Chu X Fan Z Chen L Wang H Wang P . Macrophage transition to a myofibroblast state drives fibrotic disease in uropathogenic E. coli-induced epididymo-orchitis. J Clin Investigation. (2025) 135(19):e193793. doi: 10.1172/JCI193793, PMID: 41031892 PMC12483606

[8] Min Z Wan J Xin H Liu X Rao X Fan Z . NR2C2 of macrophages promotes inflammation via NF-κB in LPS-induced orchitis in mice. Reprod (Cambridge England). (2023) 166:209–20. doi: 10.1530/REP-23-0041, PMID: 37427695

[9] Hasan H Peng W Wijayarathna R Wahle E Fietz D Bhushan S . Monocytes expressing activin A and CCR2 exacerbate chronic testicular inflammation by promoting immune cell infiltration. Hum Reprod (Oxford England). (2024). doi: 10.1093/humrep/deae107, PMID: 38775335

[10] Palsson-McDermott EM Curtis AM Goel G Lauterbach MAR Sheedy FJ Gleeson LE . Pyruvate kinase M2 regulates Hif-1α activity and IL-1β induction and is a critical determinant of the warburg effect in LPS-activated macrophages. Cell Metab. (2015) 21:65–80. doi: 10.1016/j.cmet.2014.12.005, PMID: 25565206 PMC5198835

[11] Feng T Zhao X Gu P Yang W Wang C Guo Q . Adipocyte-derived lactate is a signalling metabolite that potentiates adipose macrophage inflammation via targeting PHD2. Nat Commun. (2022) 13:5208. doi: 10.1038/s41467-022-32871-3, PMID: 36064857 PMC9445001

[12] Peng M Yin N Chhangawala S Xu K Leslie CS Li MO . Aerobic glycolysis promotes T helper 1 cell differentiation through an epigenetic mechanism. Science. (2016) 354:481–4. doi: 10.1126/science.aaf6284, PMID: 27708054 PMC5539971

[13] Colegio OR Chu NQ Szabo AL Chu T Rhebergen AM Jairam V . Functional polarization of tumour-associated macrophages by tumour-derived lactic acid. Nature. (2014) 513:559–63. doi: 10.1038/nature13490, PMID: 25043024 PMC4301845

[14] Zhang D Tang Z Huang H Zhou G Cui C Weng Y . Metabolic regulation of gene expression by histone lactylation. Nature. (2019) 574:575–80. doi: 10.1038/s41586-019-1678-1, PMID: 31645732 PMC6818755

[15] Irizarry-Caro RA McDaniel MM Overcast GR Jain VG Troutman TD Pasare C . TLR signaling adapter BCAP regulates inflammatory to reparatory macrophage transition by promoting histone lactylation. Proc Natl Acad Sci United States America. (2020) 117:30628–38. doi: 10.1073/pnas.2009778117, PMID: 33199625 PMC7720107

[16] Yang K Fan M Wang X Xu J Wang Y Tu F . Lactate promotes macrophage HMGB1 lactylation, acetylation, and exosomal release in polymicrobial sepsis. Cell Death Differentiation. (2021) 29:133–46. doi: 10.1038/s41418-021-00841-9, PMID: 34363018 PMC8738735

[17] Xie B Zhang M Li J Cui J Zhang P Liu F . KAT8-catalyzed lactylation promotes eEF1A2-mediated protein synthesis and colorectal carcinogenesis. Proc Natl Acad Sci United States America. (2024) 121:e2314128121. doi: 10.1073/pnas.2314128121, PMID: 38359291 PMC10895275

[18] Klein B Bhushan S Günther S Middendorff R Loveland KL Hedger MP . Differential tissue-specific damage caused by bacterial epididymo-orchitis in the mouse. Mol Hum Reprod. (2020) 26:215–27. doi: 10.1093/molehr/gaaa011, PMID: 32011693 PMC7187874

[19] Zeng R Jin C Zheng C Li S Qian S Pan J . OCT4 represses inflammation and cell injury during orchitis by regulating CIP2A expression. Front In Cell Dev Biology. (2021) 9:683209. doi: 10.3389/fcell.2021.683209, PMID: 34513828 PMC8427512

[20] Ma C Huang J Jiang Y Liu L Wang N Huang S . Gasdermin D in macrophages drives orchitis by regulating inflammation and antigen presentation processes. EMBO Mol Med. (2024) 16:361–85. doi: 10.1038/s44321-023-00016-8, PMID: 38177538 PMC10897472

[21] Wilharm A Brigas HC Sandrock I Ribeiro M Amado T Reinhardt A . Microbiota-dependent expansion of testicular IL-17-producing Vγ6+ γδ T cells upon puberty promotes local tissue immune surveillance. Mucosal Immunol. (2021) 14:242–52. doi: 10.1038/s41385-020-0330-6, PMID: 32733025 PMC7790758

[22] Sun T Liu B Li Y Wu J Cao Y Yang S . Oxamate enhances the efficacy of CAR-T therapy against glioblastoma via suppressing ectonucleotidases and CCR8 lactylation. J Exp Clin Cancer Research: CR. (2023) 42:253. doi: 10.1186/s13046-023-02815-w, PMID: 37770937 PMC10540361

[23] Rho H Terry AR Chronis C Hay N . Hexokinase 2-mediated gene expression via histone lactylation is required for hepatic stellate cell activation and liver fibrosis. Cell Metab. (2023) 35(8):1406–23.e8. doi: 10.1016/j.cmet.2023.06.013, PMID: 37463576 PMC11748916

[24] Jandl K Marsh LM Mutgan AC Crnkovic S Valzano F Zabini D . Impairment of the NKT-STAT1-CXCL9 axis contributes to vessel fibrosis in pulmonary hypertension caused by lung fibrosis. Am J Respir Crit Care Med. (2022) 206:981–98. doi: 10.1164/rccm.202201-0142OC, PMID: 35763380

[25] Shang S Yang Y-W Chen F Yu L Shen S-H Li K . TRIB3 reduces CD8+ T cell infiltration and induces immune evasion by repressing the STAT1-CXCL10 axis in colorectal cancer. Sci Trans Med. (2022) 14:eabf0992. doi: 10.1126/scitranslmed.abf0992, PMID: 34985967

[26] Tong M Smeekens JM Xiao H Wu R . Systematic quantification of the dynamics of newly synthesized proteins unveiling their degradation pathways in human cells. Chem Sci. (2020) 11:3557–68. doi: 10.1039/c9sc06479f, PMID: 34109028 PMC8152571

[27] Huang H Tsui Y-M Ho DW-H Chung CY-S Sze KM-F Lee E . LANCL1, a cell surface protein, promotes liver tumor initiation through FAM49B-Rac1 axis to suppress oxidative stress. Hepatol (Baltimore Md.). (2024) 79:323–40. doi: 10.1097/HEP.0000000000000523, PMID: 37540188 PMC10789379

[28] Li D Wu R Guo W Xie L Qiao Z Chen S . STING-mediated IFI16 degradation negatively controls type I interferon production. Cell Rep. (2019) 29(5):1249–60.e4. doi: 10.1016/j.celrep.2019.09.069, PMID: 31665637

[29] Li Z Huan C Wang H Liu Y Liu X Su X . TRIM21-mediated proteasomal degradation of SAMHD1 regulates its antiviral activity. EMBO Rep. (2020) 21:e47528. doi: 10.15252/embr.201847528, PMID: 31797533 PMC6944907

[30] Gu J Zhou J Chen Q Xu X Gao J Li X . Tumor metabolite lactate promotes tumorigenesis by modulating MOESIN lactylation and enhancing TGF-β signaling in regulatory T cells. Cell Rep. (2022) 39:110986. doi: 10.1016/j.celrep.2022.110986, PMID: 35732125

[31] Gao W Li Y Liu X Wang S Mei P Chen Z . TRIM21 regulates pyroptotic cell death by promoting Gasdermin D oligomerization. Cell Death Differentiation. (2022) 29:439–50. doi: 10.1038/s41418-021-00867-z, PMID: 34511601 PMC8817046

[32] Lugassy J Abdala-Saleh N Jarrous G Turky A Saidemberg D Ridner-Bahar G . Development of DPP-4-resistant CXCL9-Fc and CXCL10-Fc chemokines for effective cancer immunotherapy. Proc Natl Acad Sci United States America. (2025) 122:e2501791122. doi: 10.1073/pnas.2501791122, PMID: 40238455 PMC12037015

[33] Jiang Q Wang F Shi L Zhao X Gong M Liu W . C-X-C motif chemokine ligand 10 produced by mouse Sertoli cells in response to mumps virus infection induces male germ cell apoptosis. Cell Death Dis. (2017) 8:e3146. doi: 10.1038/cddis.2017.560, PMID: 29072682 PMC5680925

[34] Flier J Boorsma DM van Beek PJ Nieboer C Stoof TJ Willemze R . Differential expression of CXCR3 targeting chemokines CXCL10, CXCL9, and CXCL11 in different types of skin inflammation. J Pathol. (2001) 194:398–405. doi: 10.1002/1096-9896(200108)194:4<397::AID-PATH899>3.0.CO;2-S, PMID: 11523046

[35] Seles M Altziebler J Gorkiewicz G Kriegl L Hatzl S Ahyai S . Human Tularemia Epididymo-Orchitis Caused by Francisella tularensis Subspecies holartica, Austria. Emerging Infect Dis. (2023) 29:2105–7. doi: 10.3201/eid2910.230436, PMID: 37735772 PMC10521599

[36] Gardini G Comelli A Pecorelli S Parolini F Tomasoni L Pezzotta R . Acute epididymo-orchitis due to Salmonella Typhi in a young man from Bangladesh. Infection. (2019) 47:857–60. doi: 10.1007/s15010-019-01280-y, PMID: 30771193

[37] Wang F Liu W Jiang Q Gong M Chen R Wu H . Lipopolysaccharide-induced testicular dysfunction and epididymitis in mice: a critical role of tumor necrosis factor alpha†. Biol Reprod. (2019) 100:849–61. doi: 10.1093/biolre/ioy235, PMID: 30398566

[38] Fan ZP Peng ML Chen YY Xia YZ Liu CY Zhao K . S100A9 activates the immunosuppressive switch through the PI3K/akt pathway to maintain the immune suppression function of testicular macrophages. Front In Immunol. (2021) 12:743354. doi: 10.3389/fimmu.2021.743354, PMID: 34764959 PMC8576360

[39] Beck-Schimmer B Schwendener R Pasch T Reyes L Booy C Schimmer RC . Alveolar macrophages regulate neutrophil recruitment in endotoxin-induced lung injury. Respir Res. (2005) 6:61. doi: 10.1186/1465-9921-6-61, PMID: 15972102 PMC1188075

[40] Gaytan F Bellido C Morales C Reymundo C Aguilar E van Rooijen N . Response to Leydig cell apoptosis in the absence of testicular macrophages. J Reprod Immunol. (1995) 29:81–94. doi: 10.1016/0165-0378(95)00934-D, PMID: 8531194

[41] Lu Z Li X Yang P Mu G He L Song C . Heparin-binding protein enhances NF-κB pathway-mediated inflammatory gene transcription in M1 macrophages via lactate. Inflammation. (2021) 44:48–56. doi: 10.1007/s10753-020-01263-4, PMID: 33052541

[42] Cai H Chen X Liu Y Chen Y Zhong G Chen X . Lactate activates trained immunity by fueling the tricarboxylic acid cycle and regulating histone lactylation. Nat Commun. (2025) 16:3230. doi: 10.1038/s41467-025-58563-2, PMID: 40185732 PMC11971257

[43] Sun S Xu X Liang L Wang X Bai X Zhu L . Lactic Acid-Producing Probiotic Saccharomyces cerevisiae Attenuates Ulcerative Colitis via Suppressing Macrophage Pyroptosis and Modulating Gut Microbiota. Front Immunol. (2021) 12:777665. doi: 10.3389/fimmu.2021.777665, PMID: 34899735 PMC8652295

[44] Liu J Zhang C Xu D Zhang T Chang C-Y Wang J . The ubiquitin ligase TRIM21 regulates mutant p53 accumulation and gain of function in cancer. J Clin Invest. (2023) 133(6):e164354. doi: 10.1172/JCI164354, PMID: 36749630 PMC10014102

[45] Hagihara H Shoji H Otabi H Toyoda A Katoh K Namihira M . Protein lactylation induced by neural excitation. Cell Rep. (2021) 37:109820. doi: 10.1016/j.celrep.2021.109820, PMID: 34644564

[46] Gu J Zhou J Chen Q Xu X Gao J Li X . Tumor metabolite lactate promotes tumorigenesis by modulating MOESIN lactylation and enhancing TGF-beta signaling in regulatory T cells. Cell Rep. (2022) 39:110986. doi: 10.1016/j.celrep.2022.110986, PMID: 35732125

[47] Wang J Yang P Yu T Gao M Liu D Zhang J . Lactylation of PKM2 suppresses inflammatory metabolic adaptation in pro-inflammatory macrophages. Int J Biol Sci. (2022) 18:6210–25. doi: 10.7150/ijbs.75434, PMID: 36439872 PMC9682528

[48] Yang K Fan M Wang X Xu J Wang Y Tu F . Lactate promotes macrophage HMGB1 lactylation, acetylation, and exosomal release in polymicrobial sepsis. Cell Death Differentiation. (2022) 29:133–46. doi: 10.1038/s41418-021-00841-9, PMID: 34363018 PMC8738735

[49] Qin Q Wang D Qu Y Li J An K Mao Z . Enhanced glycolysis-derived lactate promotes microglial activation in Parkinson’s disease via histone lactylation. NPJ Parkinson’s Dis. (2025) 11:3. doi: 10.1038/s41531-024-00858-0, PMID: 39753581 PMC11698869

[50] Sun L Zhang Y Yang B Sun S Zhang P Luo Z . Lactylation of METTL16 promotes cuproptosis via m6A-modification on FDX1 mRNA in gastric cancer. Nat Commun. (2023) 14:6523. doi: 10.1038/s41467-023-42025-8, PMID: 37863889 PMC10589265

[51] Shiota H Barral S Buchou T Tan M Couté Y Charbonnier G . Nut directs p300-dependent, genome-wide H4 hyperacetylation in male germ cells. Cell Rep. (2018) 24(13):3477–87.e6. doi: 10.1016/j.celrep.2018.08.069, PMID: 30257209

[52] Tatone C Di Emidio G Barbonetti A Carta G Luciano AM Falone S . Sirtuins in gamete biology and reproductive physiology: emerging roles and therapeutic potential in female and male infertility. Hum Reprod Update. (2018) 24:267–89. doi: 10.1093/humupd/dmy003, PMID: 29447380

[53] Li H Hu Y-F Wang X-R Ouyang K-W Wang H Wang K-W . Suppressed testicular macrophage M1 polarization by HDAC5 enforces insensitivity to LPS-elicited blood-testis barrier damage. Food Chem Toxicology: an Int J Published For Br Ind Biol Res Assoc. (2024) 192:114940. doi: 10.1016/j.fct.2024.114940, PMID: 39151879

[54] Wijayarathna R de Geus ED Genovese R Gearing LJ Wray-McCann G Sreenivasan R . Interferon epsilon is produced in the testis and protects the male reproductive tract against virus infection, inflammation and damage. PloS Pathog. (2024) 20:e1012702. doi: 10.1371/journal.ppat.1012702, PMID: 39621805 PMC11637430

[55] Wang M Yang Y Cansever D Wang Y Kantores C Messiaen S . Two populations of self-maintaining monocyte-independent macrophages exist in adult epididymis and testis. Proc Natl Acad Sci U S A. (2021) 118(1):e2013686117. doi: 10.1073/pnas.2013686117, PMID: 33372158 PMC7817195

[56] Guo L Qin T-Z Liu L-Y Lai P-P Xue Y-Z Jing Y-T . The abscopal effects of cranial irradiation induce testicular damage in mice. Front In Physiol. (2021) 12:717571. doi: 10.3389/fphys.2021.717571, PMID: 34867437 PMC8637864

[57] Jing Y Cao M Zhang B Long X Wang X . cDC1 dependent accumulation of memory T cells is required for chronic autoimmune inflammation in murine testis. Front In Immunol. (2021) 12:651860. doi: 10.3389/fimmu.2021.651860, PMID: 34381443 PMC8350123

