# Supplementary figures and images for: Macrophage TRIM21 lactylation exacerbates infection-induced orchitis through enhancing STAT1-mediated CXCL9 and CXCL10 production
Source: Front Immunol. 2026 Jan 14;16:1684836. doi: 10.3389/fimmu.2025.1684836 (PMC12847007; doi:10.3389/fimmu.2025.1684836)

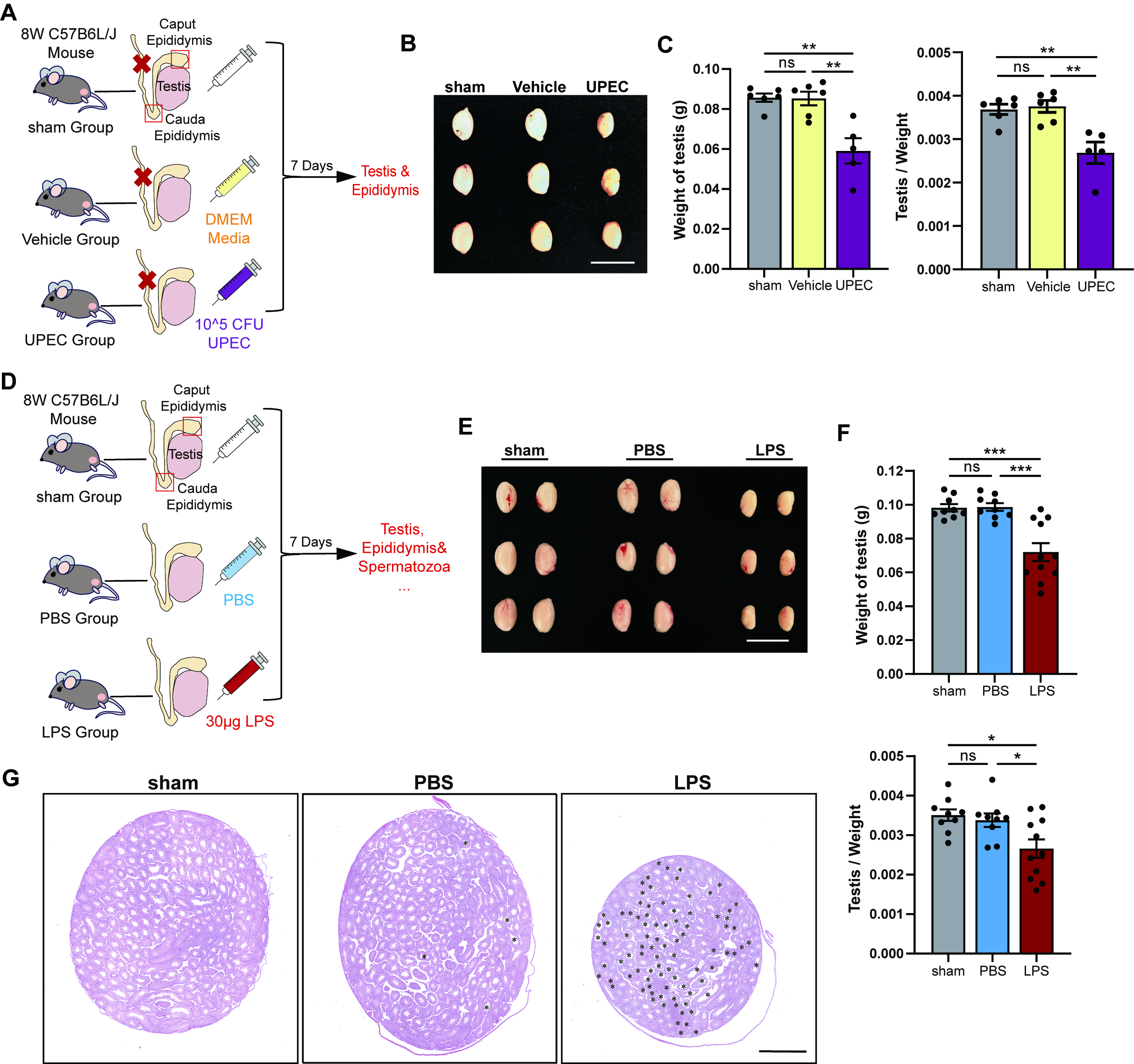

Supplement: Supplementary file 1 [file Image1.tif]

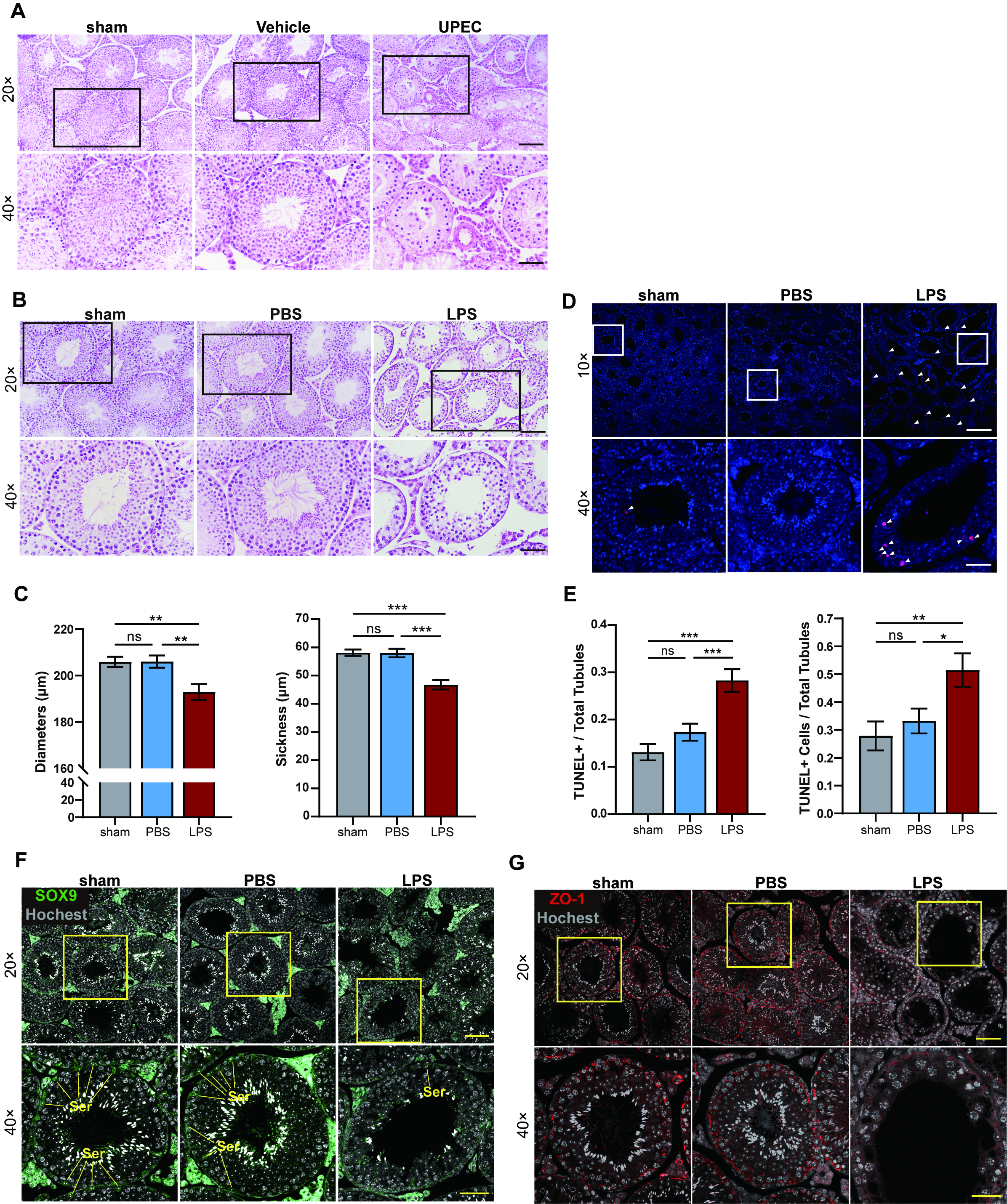

Supplement: Supplementary file 2 [file Image2.tif]

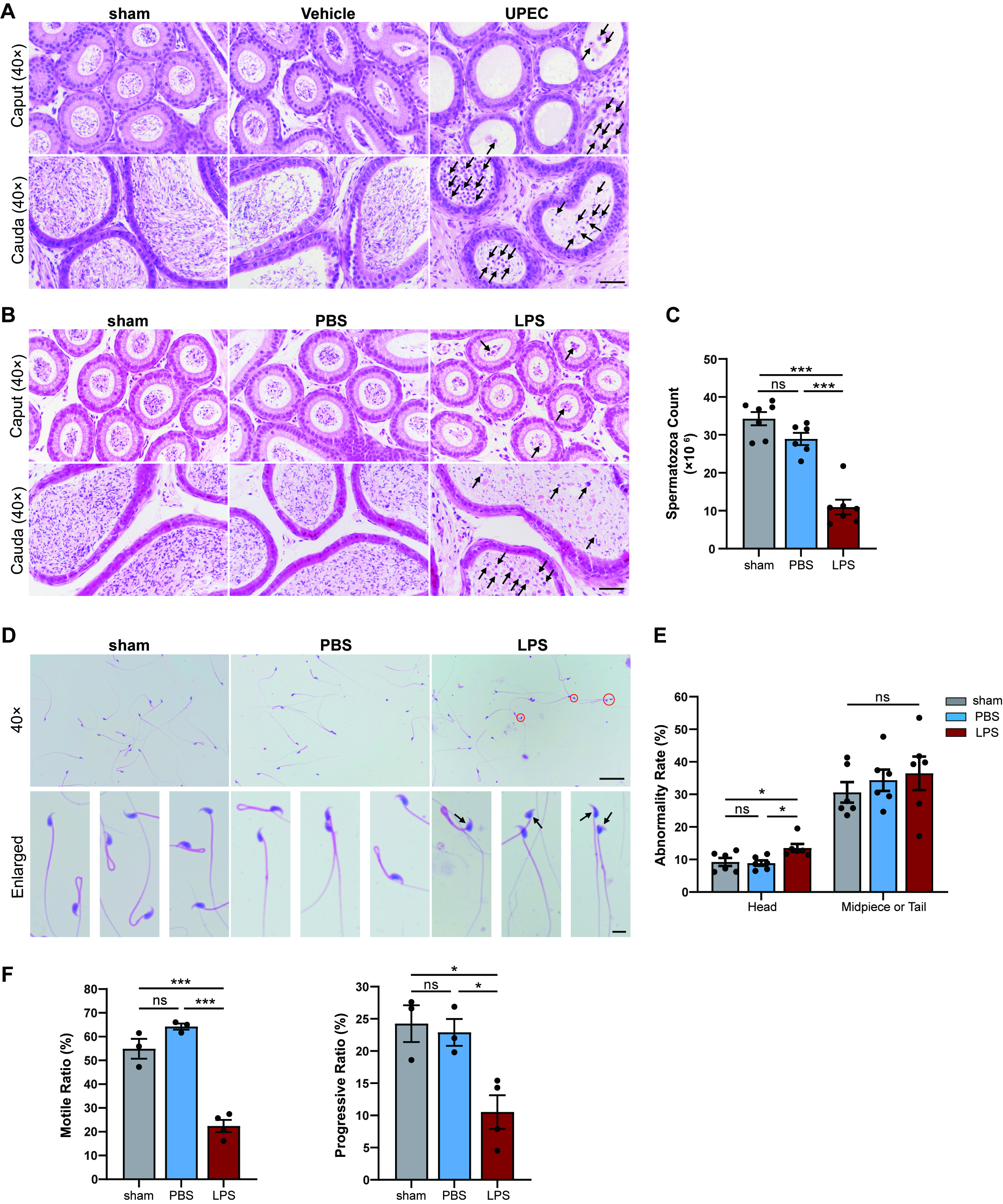

Supplement: Supplementary file 3 [file Image3.tif]

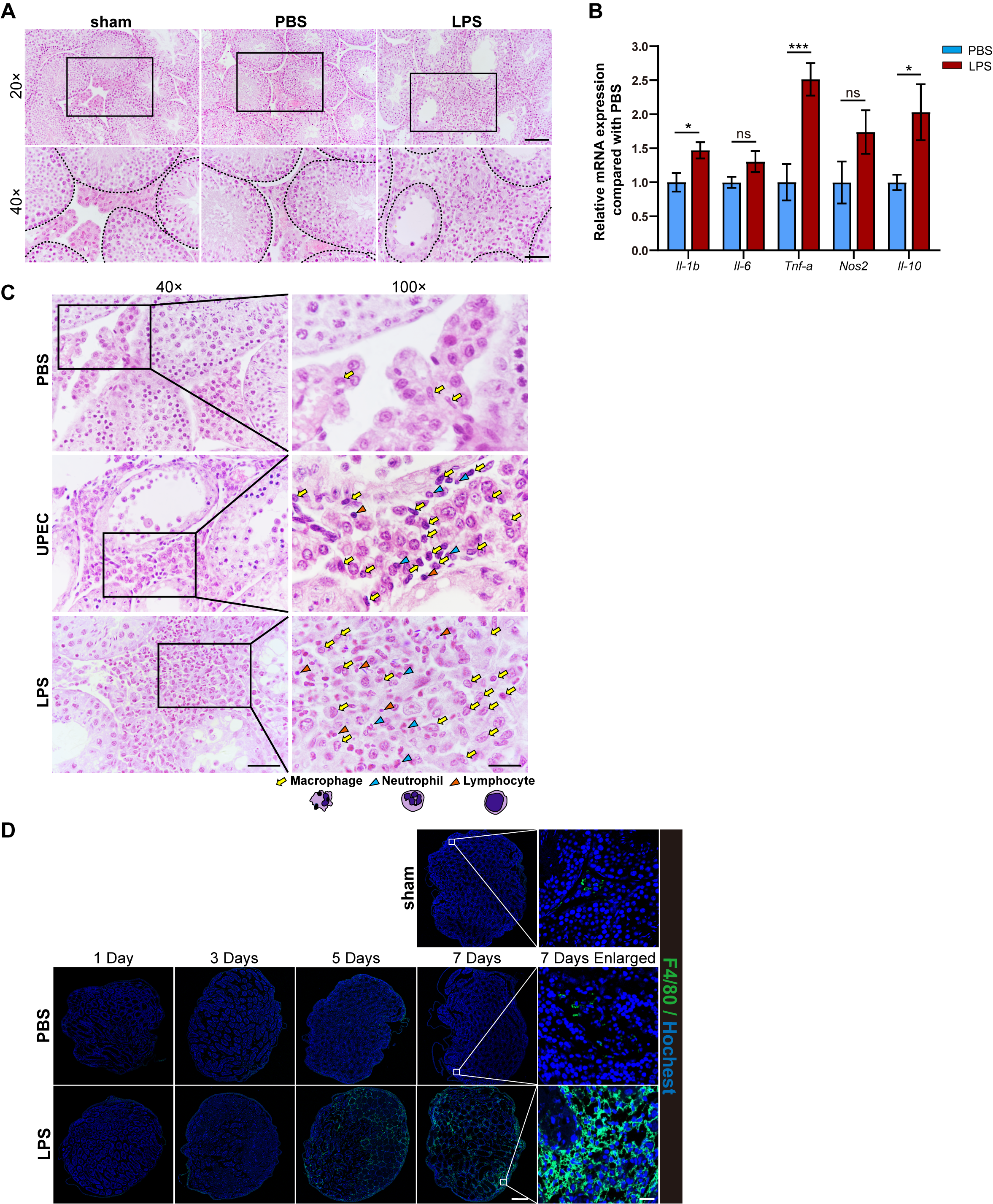

Supplement: Supplementary file 4 [file Image4.tif]

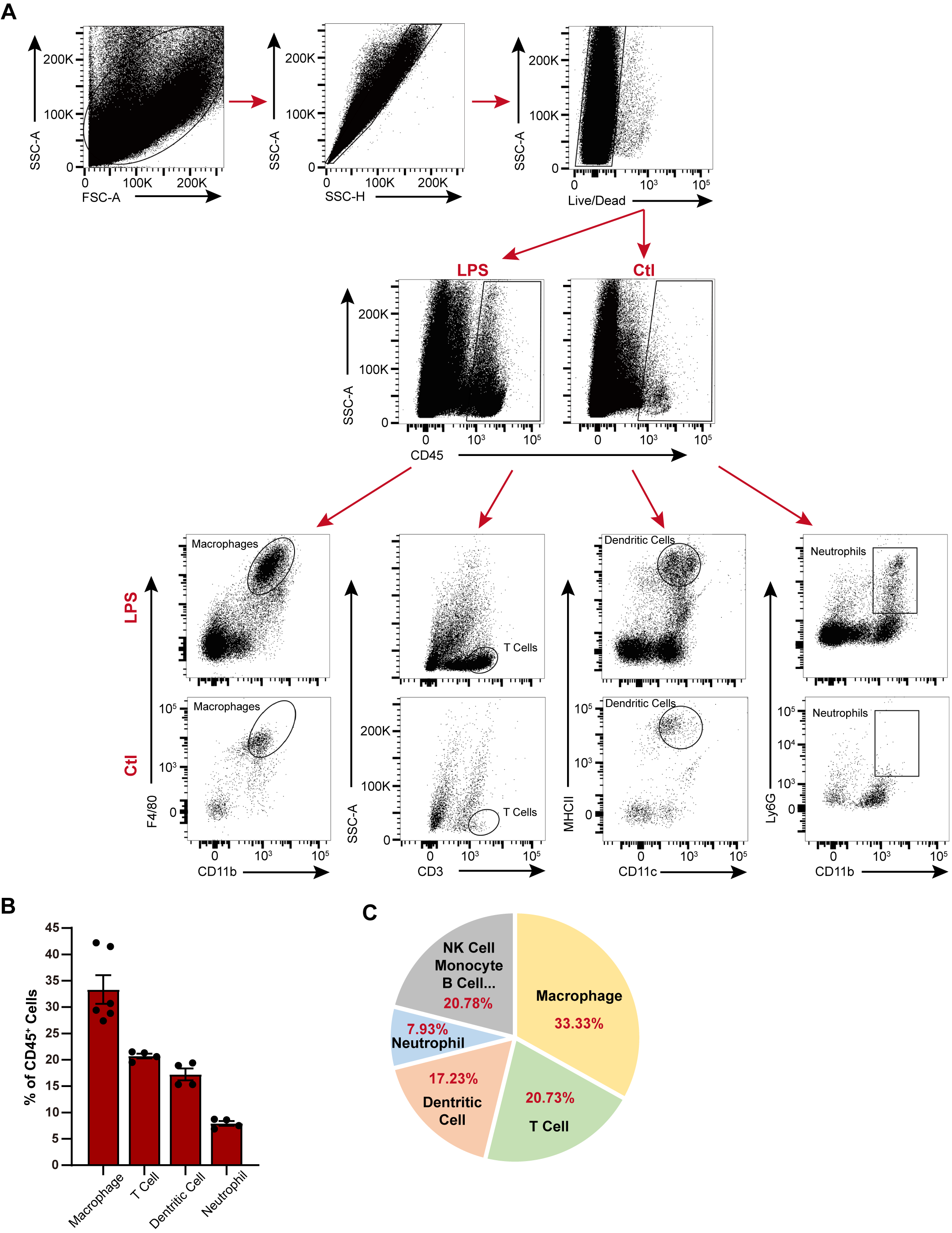

Supplement: Supplementary file 5 [file Image5.tif]

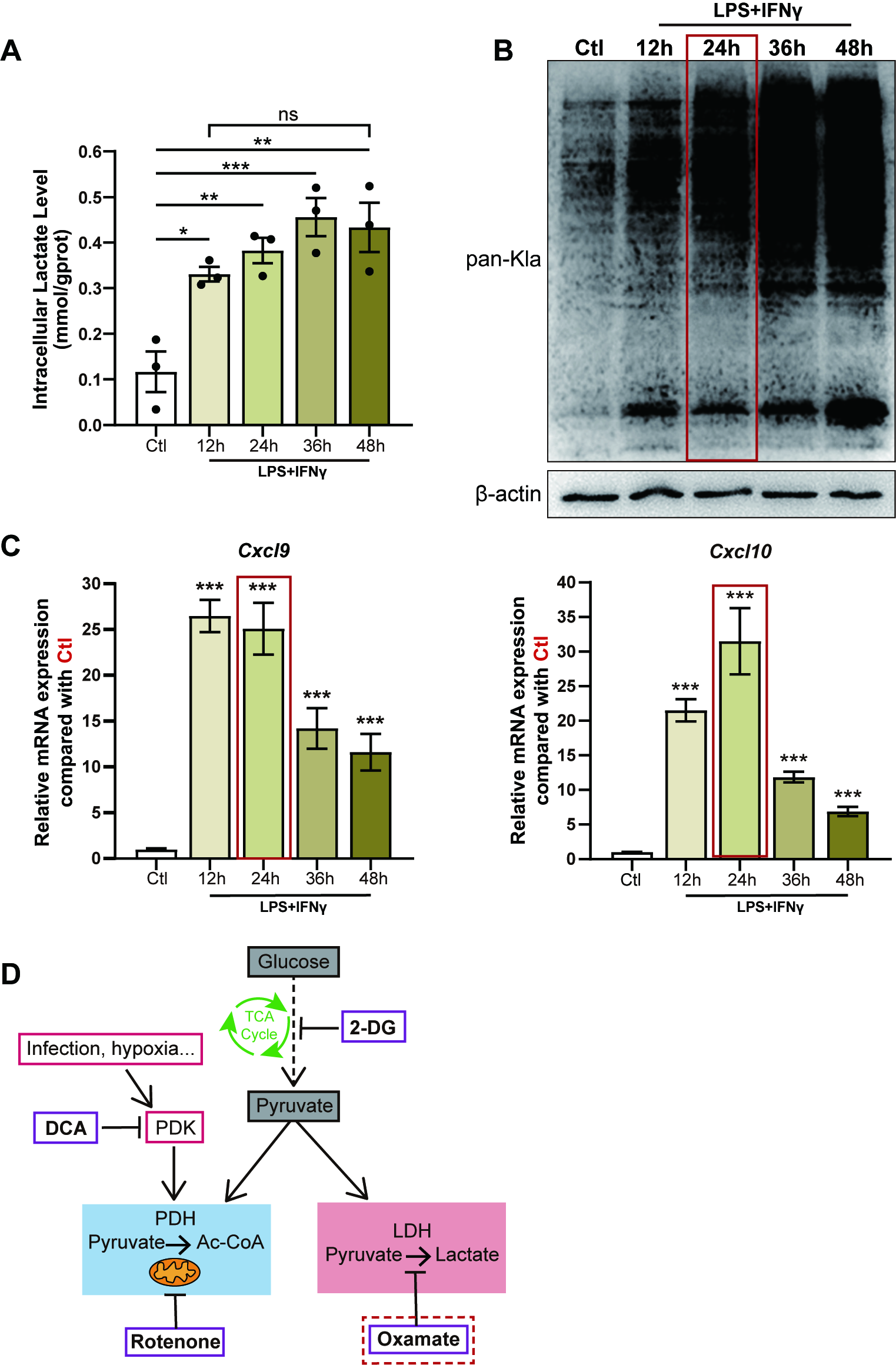

Supplement: Supplementary file 6 [file Image6.tif]
