# Supplementary material for: Macrophage TRIM21 lactylation exacerbates infection-induced orchitis through enhancing STAT1-mediated CXCL9 and CXCL10 production
Source: Front Immunol. 2026 Jan 14;16:1684836. doi: 10.3389/fimmu.2025.1684836 (PMC12847007; doi:10.3389/fimmu.2025.1684836)
Supplement: Supplementary file 7 [file Supplementaryfile1.pdf]

Supplementary Figure legends

Supplemental Figure 1. *In situ* injection of UPEC or lipopolysaccharid could successfully induce orchitis in mice.

(A) Schematic diagram showing the operations conducted on sham / vehicle / UPEC group adult C57B6L/J mice. The red cross at the vas deferens indicates ligation to prevent the spread of uropathogenic *Escherichia coli* through the urogenital tract. (B) Gross morphology of representative testes from UPEC-induced orchitic mice, vehicle control and sham group mice. Scale bar, 1 cm. (C) Weight and relative weight of testes from sham / vehicle / UPEC-induced orchitic mice (n = 5-6). (D) Schematic diagram showing the operations conducted on sham/ PBS/ LPS group adult C57B6L/J mice. (E) Gross morphology of representative testes from LPS-induced orchitic mice, PBS control and sham group mice. Scale bar, 1 cm. (F) Weight and relative weight of sham / PBS / LPS-induced orchitic mice (n = 10-11). (G) H&E staining of the cross-sections from testes of sham / PBS / LPS-induced orchitic mice. Asterisks (*) indicate impaired seminiferous tubules with empty lumens. Data are statistically compared with one-way ANOVA. Error bars represent mean ± SEM. *$p < 0.05$, **$p < 0.01$, ***$p < 0.001$, ns, not significant.

Supplemental Figure 2. Infection-induced orchitis impaired spermatogenesis and blood-testis barrier in mice.

(A) Representative H&E staining images of seminiferous tubules from testes of sham / vehicle / UPEC-induced orchitic mice. Scale bars, 100 μm at 20× magnification, 50 μm at 40× magnification. (B) Representative H&E staining images of seminiferous tubules from

testes of sham / PBS / LPS-induced orchitic mice. Scale bars, 100 μm at 20× magnification, 50 μm at 40× magnification. (C) Quantification of seminiferous tubule diameter and seminiferous epithelium thickness of representative seminiferous tubules in testes from sham / PBS / LPS-induced orchitic mice (n = 3). (D-E) Representative images and quantification of TUNEL apoptosis assay in testes from sham / PBS / LPS-induced orchitic mice (n = 3). White arrowheads indicate apoptotic germ cells with positive TUNEL fluorescent signal. Scale bars, 200 μm at 10× magnification, 50 μm at 40× magnification. (F-G) Representative immunofluorescence images showing the expression of Sertoli cell-specific SOX9 and tight junction protein ZO-1 in typical seminiferous tubules of testes from sham / PBS / LPS-induced orchitic mice (Ser, Sertoli Cell). Scale bars, 100 μm at 20× magnification, 50 μm at 40× magnification. Data are statistically compared with one-way ANOVA. Error bars represent mean ± SEM. *$p < 0.05$, **$p < 0.01$, ***$p < 0.001$, ns, not significant.

Supplemental Figure 3. The number and quality of spermatozoa cells were decreased in mice with orchtis.

(A) Representative H&E staining images of caput epididymis and cauda epididymis from sham / vehicle / UPEC-induced orchitic mice. Black arrows indicate dead cells in the lumens of caput epididymis and cauda epididymis. Scale bar, 50 μm at 40× magnification. (B) Representative H&E staining images of caput epididymis and cauda epididymis from sham / PBS / LPS-induced orchitic mice. Black arrows indicate dead cells in the lumens of caput epididymis and cauda epididymis. Scale bar, 50 μm at 40× magnification. (C) Spermatozoa count in cauda epididymis of sham / PBS / LPS-induced orchitic mice (n = 6-7). (D)

Representative H&E staining images of spermatozoa from sham / PBS / LPS-induced orchitic mice. Black arrows indicate abnormal spermatozoa (head abnormality). Scale bar, 50 μm at 40× magnification, 2 μm in enlarged images. (E) Head abnormality rate and midpiece or tail abnormality rate of spermatozoa from sham / PBS / LPS-induced orchitic mice (n = 6). (F) Ratio of motile and progressive spermatozoa of sham / PBS / LPS-induced orchitic mice (n = 3-4). Data are statistically compared with one-way ANOVA. Error bars represent mean ± SEM. *$p < 0.05$, ***$p < 0.001$, ns, not significant.

Supplemental Figure 4. Macrophages were the majority of infiltrated inflammatory cells in the testis during LPS-induced orchitis.

(A) H&E staining images showing the immune infiltration in testicular interstitium from sham/ PBS/ LPS-induced orchitic mice. Dotted lines indicate the edge of seminiferous tubules. Scale bars, 100 μm at 20× magnification, 50 μm at 40× magnification. (B) Relative mRNA levels in the testes from LPS-induced orchitic and PBS control mice (n = 3). (C) Representative images of H&E staining of testicular interstitium from the indicated mice treated with PBS/ UPEC/ LPS for 7 days. Different arrows indicate different immune cells as shown. Scale bars, 50 μm at 40× magnification, 20 μm at 100× magnification. (D) Scan montage showing the distribution of F4/80-positive macrophages in whole testis 1, 3, 5, and 7 days after PBS/ LPS injection, with normal testis as negative control. Scale bar, 500 μm, 20 μm in 100× enlarged images. Data are statistically compared with Student *t*-test. Error bars represent mean ± SEM. *$p < 0.05$, ***$p < 0.001$, ns, not significant.

Supplemental Figure 5. The flow-cytometry gating strategy used to define immune cell subsets. (A) Representative FACS plots showing the gating strategies for $CD45^+$ cells, Macrophages ($CD11b^+F4/80^+$), T cells ($CD3^+$), dendritic cells ($CD11c^+MHCII^+$), and neutrophils ($CD11b^+Ly6G^+$) from the testis of LPS-induced orchitis and control mice (pre-gated on single cell and live cell). (B) Quantified flow cytometry of macrophage ($CD11b^+F4/80^+$), T cell ($CD3^+$), dentritic cell ($CD11c^+MHCII^+$) and neutrophil ($CD11b^+Ly6G^+$) in whole testes from LPS-induced orchitic mice (gated on $CD45^+$ cells, n = 3). (C) Overall composition of aforementioned immune cells in the testes from LPS-induced orchitic mice (n = 3).

Supplemental Figure 6. The levels of intracellular lactate, lactylation CXCL9, and CXCL10 were increased in macrophages treated with LPS and IFN-γ.

(A) Intracellular lactate levels in Raw264.7 cells within 48 hours after stimulation with LPS + IFN-γ (n = 3, normalized with intracellular protein levels). Raw264.7 cells without LPS + IFN-γ stimulation served as control (Ctl). (B) Global lysine lactylation levels in Raw264.7 cells within 48 hours after stimulation with LPS + IFN-γ. (C) Relative mRNA levels of *Cxcl9* and *Cxcl10* in Raw264.7 cells of groups mentioned above (n = 3). Asterisks indicate comparison with the Ctl group. (D) Schematic comparison of oxamate (indicated by red dashed box) with other glycolysis/oxidative phosphorylation inhibitors (indicated by purple boxes). 2-DG: 2-deoxy-D-glucose, hexokinase inhibitor; DCA: dichloroacetate, pyruvate dehydrogenase kinase (PDK) inhibitor; Rotenone: mitochondrial complex I inhibitor. Data are statistically compared with one-way ANOVA. Error bars represent mean ± SEM. *$p < 0.05$,

**$p < 0.01$, ***$p < 0.001$, ns, not significant.
